# SIRT4 Promotes Pancreatic Cancer Stemness by Enhancing Histone Lactylation and Epigenetic Reprogramming Stimulated by Calcium Signaling

**DOI:** 10.1002/advs.202412553

**Published:** 2025-04-29

**Authors:** Mengzhu Lv, Xiaodan Yang, Congcong Xu, Qingru Song, Hailian Zhao, Tianjiao Sun, Jingtao Liu, Yuan Zhang, Guogui Sun, Yuanchao Xue, Zhiqian Zhang

**Affiliations:** ^1^ Key Laboratory of Carcinogenesis and Translational Research (Ministry of Education), Department of Cell Biology Peking University Cancer Hospital & Institute Beijing 100142 P. R. China; ^2^ State Key Laboratory of Metabolic Dysregulation & Prevention and Treatment of Esophageal Cancer The First Affiliated Hospital of Zhengzhou University Zhengzhou Henan 450052 P. R. China; ^3^ Key Laboratory of RNA Biology, Institute of Biophysics Chinese Academy of Sciences Beijing 100101 P. R. China; ^4^ Key Laboratory of Carcinogenesis and Translational Research (Ministry of Education), Department of Pharmacy Peking University Cancer Hospital and Institute Beijing 100142 P. R. China; ^5^ Department of Chemoradiation Affiliated Hospital of North China University of Science and Technology Tangshan Hebei 063000 P.R. China

**Keywords:** glycolysis, histones lactylation, SIRT4, tumor‐initiating cells

## Abstract

Mitochondria Sirtuins including SIRT4 erase a variety of posttranslational modifications from mitochondria proteins, leading to metabolic reprogramming that acts as a tumor suppressor, oncogenic promotor, or both. However, the factors and the underlying mechanisms that stimulate and relay such a signaling cascade are poorly understood. Here, we reveal that the voltage‐gated calcium channel subunit α2δ1‐mediated calcium signaling can upregulate the expression of SIRT4, which is highly expressed in α2δ1‐positive pancreatic tumor‐initiating cells (TICs). Furthermore, SIRT4 is functionally sufficient and indispensable to promote TIC properties of pancreatic cancer cells by directly deacetylating ENO1 at K358, leading to attenuated ENO1's RNA‐binding capacity, enhanced glycolytic substrate 2‐PG affinity, and subsequently robust catalytic activity with boosted glycolytic ability and increased production of lactate acid. Interestingly, both SIRT4 and deacetylated mimetic of ENO1‐K358 can increase the lactylation of histones at multiple sites including H3K9 and H3K18 sites, which resulted in epigenetic reprogramming to directly activate a variety of pathways that are essential for stemness. Hence, the study links α2δ1‐mediated calcium signaling to SIRT4‐mediated histone lactylation epigenetic reprogramming in promoting the stem cell‐like properties of pancreatic cancer, which holds significant potential for the development of novel therapeutic strategies by targeting TICs of pancreatic cancer.

## Graphical abstract

As a downstream target of α2δ1‐mediated calcium signaling, SIRT4 serves as an oncogene to promote the stemness of pancreatic cancer via deacetylating ENO1 directly at K358, leading to attenuated ENO1's RNA‐binding capacity, enhanced glycolytic substrate 2‐PG binding and robust glycolytic activity to increase the level of lactate that induce histone lactylation to epigenetically regulate signaling pathways essential for stemness.

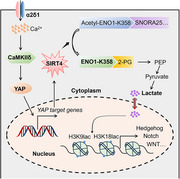

## 1 Introduction

Tumor initiating cells (TICs), also known as cancer stem cells (CSCs), are a subpopulation within a tumor that own the abilities of self‐renewal, differentiation, and generating the heterogeneous lineages of cancer cells that comprise the tumor.^[^ ^1^, ^2^ ^]^ Accumulating evidence has demonstrated that TICs are responsible for uncontrolled tumor growth, metastatic potential, resistance to conventional chemotherapy and radiotherapy, and tumor recurrence,^[^ ^3^, ^4^ ^]^ hence, therapeutic approaches targeting TICs are regarded as promising strategies to improve the outcome of cancer therapy including pancreatic ductal adenocarcinoma (PDAC), accounting for nearly 95% of pancreatic cancer, which is one of the deadly cancers with a relative 5‐year survival rate of less than 10%.^[^ ^5^, ^6^ ^]^ In PDAC, TICs have been successfully isolated with cell surface markers such as CD133, CD9, c‐Met, and DCLK1.^[^ ^7^, ^8^, ^9^ ^]^ However, TICs themselves are extremely plastic and can adapt to intrinsic and extrinsic insults or stress,^[^ ^10^ ^]^ reinforcing the need for a better understanding of the factors that mediate their plasticity and the CSC state,^[^ ^11^ ^]^ which is instrumental for developing efficient strategies of targeted therapies against PDAC TICs.

SIRT4 belongs to the Sirtuin family, a highly conserved class of nicotinamide adenine dinucleotide (NAD^+^)‐dependent lysine deacylases that remove post‐translational acyl modifications from various cellular substrates, serving as key regulators of genomic stability, metabolic homeostasis, stress response, and aging.^[^ ^12^ ^]^ In contrast to other members of Sirtuins which have a wide variety of enzymatic activities (deacetylases, decrotonylase, demalonylase, desuccinylase, ADP‐ribosyltransferases, etc.), SIRT4 represents the least characterized member in terms of enzymatic activity and function, and was originally reported with limited deacetylase activity.^[^ ^13^, ^14^ ^]^ In recent years, it has been identified as a gatekeeper of glutamine metabolism with its adenosine diphosphate (ADP)/ribosyltransferase activity,^[^ ^15^, ^16^ ^]^ a lipoamidase to inhibit pyruvate dehydrogenase complex activity,^[^ ^17^ ^]^ and a mediator of leucine metabolism and insulin secretion depending on its lysine deacylase activity.^[^ ^12^ ^]^ Interestingly, SIRT4 was later on identified with deacetylase activity. For example, It can directly deacetylate malonyl CoA decarboxylase and repress its enzymatic activity to regulate lipid metabolism,^[^ ^18^ ^]^ deacetylate and destabilize mitochondrial trifunctional protein α‐subunit (MTPα) to cause nonalcoholic fatty liver disease,^[^ ^19^ ^]^ as well as deacetylate and stabilize branched‐chain amino acid transaminase 2 (BCAT2) involved in pancreatic cancer growth.^[^ ^20^ ^]^ In addition, unlike most other Sirtuins such as SIRT1, SIRT2, and SIRT3, which appear to have dual roles in tumor suppression and promotion which are cell context‐dependent,^[^ ^21^, ^22^ ^]^ SIRT4 was overwhelmingly classified as a tumor suppressor,^[^ ^23^, ^24^, ^25^ ^]^ despite several reports indicated that SIRT4 was highly expressed and might have tumor‐promoting roles in some types of cancers.^[^ ^26^, ^27^ ^]^ Sophisticated studies addressing the tumor‐promoting roles of SIRT4 are lacking.

Enolase 1 (ENO1) is the α isoform of enolase, a glycolytic enzyme that catalyzes the conversion of 2‐phosphoglycerate (2‐PG) to phosphoenolpyruvate (PEP).^[^ ^28^ ^]^ Overexpression and/or increased enzymatic activity of ENO1 have been found in a variety of cancers including gastric, lung, and pancreatic cancers, accompanied by accelerated tumor growth and enhanced glycolysis with increased glucose uptake and lactate secretion even in the presence of oxygen, a hallmark of cancer that is called Warburg effect or aerobic glycolysis.^[^ ^29^, ^30^, ^31^, ^32^, ^33^ ^]^ Furthermore, accumulating evidence shows that posttranslational modifications of ENO1, including phosphorylation, succinylation, lysine 2‐hydroxy‐isobutyl, and phosphoglyceryl‐lysine modification, can alter its catalytic activity, localization, stability, and binding ability.^[^ ^34^, ^35^, ^36^, ^37^ ^]^ Acetylation of metabolic enzymes has been regarded as an on ⁄off switch mechanism for their activities,^[^ ^38^ ^]^ and multiple acetylated lysine residues were detected for ENO1 across a variety of cancer tissues and/or cell lines including pancreatic cancer,^[^ ^29^ ^]^ but the mechanism(s) governing the acetylation/deacetylation, and the significance of these acetylations in PDAC remain elusive.

α2δ1 is an auxiliary subunit of voltage‐gated calcium channels that mediates calcium influx into cells by functioning in both targeting and/or stabilization of the channel in the membrane and in modulating the specific gating properties of different channel‐forming subunits including the α1 subunit.^[^ ^39^, ^40^ ^]^ We and others have previously shown that the isoform 5 of α2δ1, which is specifically recognized by a monoclonal antibody 1B50‐1 (mAb1B50‐1), is a functional marker and therapeutic target for the TICs of a variety of cancers of liver, lung, gastric, and pancreatic origins.^[^ ^41^, ^42^, ^43^ ^]^ α2δ1 induced stem cell‐like properties of TICs by mediating Ca^2+^ influx, which triggers a plethora of intracellular signaling cascades including the Ca^2+^/CaMKII pathway. In this study, we reported that SIRT4, upregulated via α2δ1‐mediated calcium signaling, was an oncogenic determinant that drove the stem cell‐like properties of PDAC TICs by enhancing glycolytic activity and histone lactylation through deacetylating ENO1 at K358.

## 2 Results

### 2.1 SIRT4 Was Upregulated by α2δ1‐Triggered Calcium Signaling

**Figure 1:**
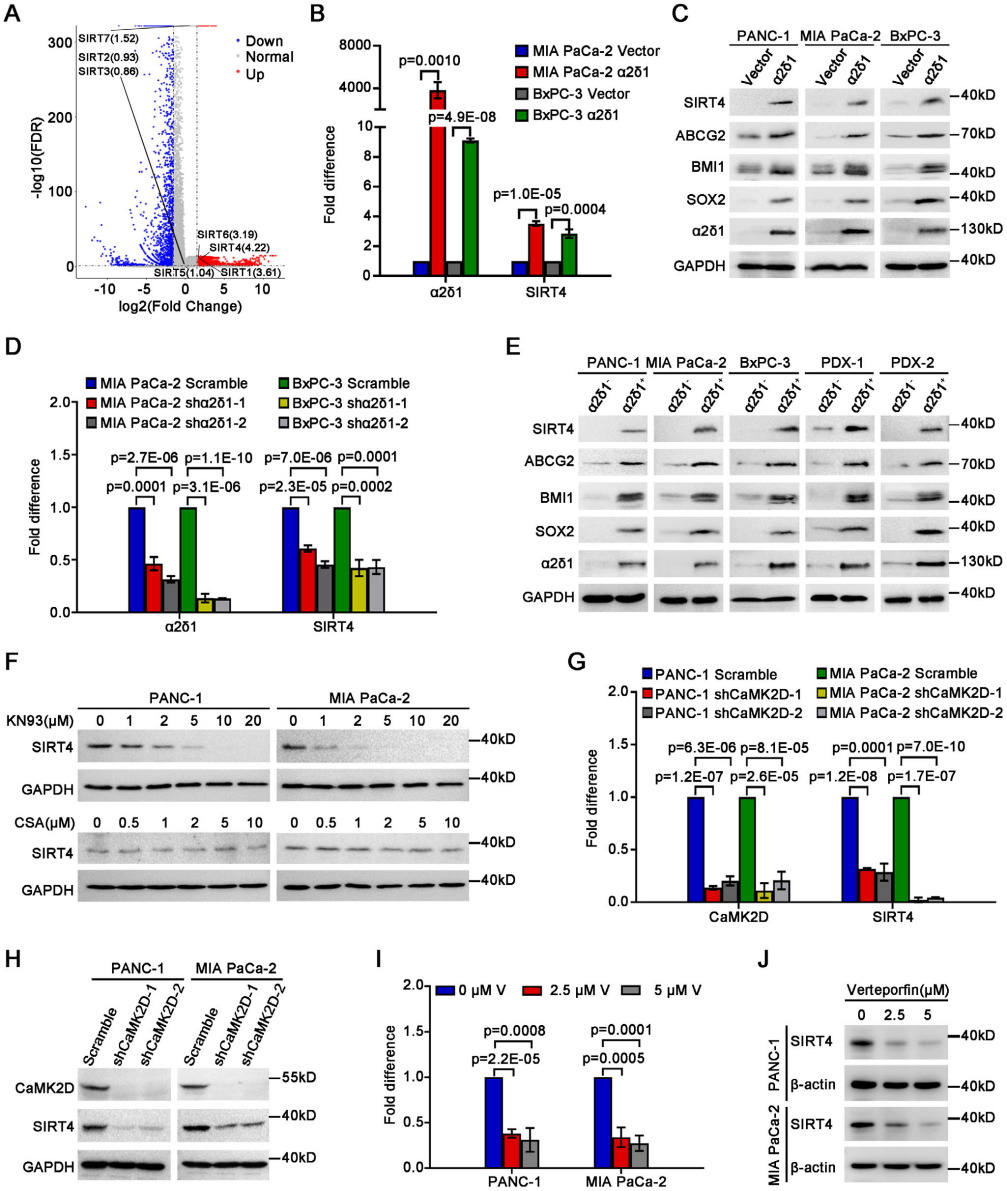
SIRT4 is upregulated by α2δ1‐triggered calcium signaling. A) Volcano plot of differentially expressed genes (|log_2_(fold change)| >1.5, FDR < 0.05) as revealed by RNA‐seq in PANC‐1 cells overexpressing α2δ1 and vector alone control. B) The expression of *SIRT4* and *α2δ1* in the indicated cells overexpressing *α2δ1* as detected by qRT‐PCR (*n* = 3). C) Western blot results demonstrating the expression of the indicated molecules in the indicated cells overexpressing α2δ1. D) The change of SIRT4 mRNA in the indicated cells after knockdown of α2δ1 with shRNAs as measured by qRT‐PCR (*n* = 3). E) Western blot analysis of the indicated molecules in the sorted α2δ1^¯^ and α2δ1^+^ fractions from the indicated sources. F) Western blot results demonstrating the expression of SIRT4 in the indicated cells treated with KN93 or cyclosporin (CsA) for 48 h. G) qRT‐PCR results showing the expression of *SIRT4* after knockdown of *CaMK2D* in the indicated cells overexpressing *α2δ1* (*n* = 3). H) Western blot analysis of SIRT4 in the indicated cells infected with lentivirus harboring *CaMK2D* shRNAs or scramble control. I) The expression of *SIRT4* in the indicated cells overexpressing *α2δ1* treated with verteporfin for 24 h as measured by qRT‐PCR (*n* = 3). J) Western blot results showing the expression of SIRT4 in the indicated cells overexpressing *α2δ1* treated with verteporfin for 24 h. β‐actin served as an internal reference in B, D, G, and I. Data were presented as mean ± SD. Unpaired two‐tailed Student's *t*‐test was used for statistical analysis.

To identify the genes regulated by α2δ1‐mediated calcium signaling in PDAC TICs, we performed RNA‐seq analysis in the PDAC cell line PANC‐1 infected with α2δ1‐overexpression (OE) or vector‐alone lentivirus. There were 4208 genes that were up‐regulated by more than two folds in the α2δ1‐OE cells, compared with the control ones. Among these upregulated genes, we were particularly interested in *SIRT4*, the most upregulated Sirtuin member (**Figure** **1**A). The upregulation of *SIRT4* by *α2δ1* was then validated in the PDAC cell lines PANC‐1, MIA PaCa‐2, and BxPC‐3 overexpressing α2δ1 by qRT‐PCR and Western blot (Figure 1B,C). On the contrary, the knockdown of *α2δ1* led to remarkable downregulation of *SIRT4* at mRNA level in α2δ1^+^ TICs purified from MIA PaCa‐2 and BxPC‐3 cells (Figure 1D). Moreover, the expression levels of SIRT4 were much higher in the α2δ1^+^ TICs sorted from these cell lines and two patient‐derived xenografts (PDX) than in the respective α2δ1‐negative (α2δ1^−^) subpopulations (Figure 1E).

We have previously demonstrated that CaMKIIδ, an isoform of the multifunctional Ca^2+^/CaM‐dependent protein kinase II (CaMKII), and Calcineurin/NFATc2 were responsible for the role of α2δ1‐mediated Ca^2+^ influx in the acquisition and maintenance of stem cell‐like properties of pancreatic and non‐small cell lung TICs, respectively.^[^ ^41^, ^42^ ^]^ To address which calcium signaling pathway was involved in the upregulation of SIRT4 mediated by α2δ1, we treated PANC‐1 and MIA PaCa‐2 cells with their inhibitors. Treatment of these cells with a CaMKII inhibitor, KN93, led to remarkable downregulation of SIRT4 in a dose‐dependent manner, whereas treatment of these cells with a calcineurin inhibitor, cyclosporin (CsA), had little effect on the expression of SIRT4 (Figure 1F), suggesting that the upregulation of SIRT4 by α2δ1‐mediated calcium signaling was mainly dependent on CaMKII, rather than on the calcineurin/NFAT pathway. Given that CaMKIIδ is the most dominantly upregulated CaMKII member by α2δ1, and is highly expressed in α2δ1^+^ PDAC TICs,^[^ ^42^ ^]^ we knocked down the expression of CaMKIIδ with its specific shRNAs in both PANC‐1 and MIA PaCa‐2 cells overexpressing α2δ1, and observed remarkable downregulation of SIRT4 (Figure 1G,H).

CaMKIIδ‐mediated phosphorylation of PKM2 at T454 and Y105 has been demonstrated to induce the nuclear localization of Yes‐associated protein (YAP) to activate the downstream YAP signaling pathway and acquire stem cell‐like properties.^[^ ^42^, ^44^ ^]^ To further address if the α2δ1–CaMKIIδ–YAP axis was indeed involved in the transcriptional upregulation of SIRT4 mediated by α2δ1, we treated both PANC‐1 and MIA PaCa‐2 cells overexpressing α2δ1 with verteporfin, a small molecule inhibitor of YAP. As expected, the promoting role of *α2δ1* on the expression of *SIRT4* was retarded significantly by verteporfin in a dose‐dependent manner (Figures 1I,J).

Collectively, these data demonstrate that SIRT4 is upregulated at both mRNA and protein levels by α2δ1‐mediated calcium signaling involved α2δ1–CaMKIIδ–YAP axis, and is highly expressed in α2δ1^+^ PDAC TICs.

### 2.2 SIRT4 Is Required for the Maintenance of Stem Cell‐Like Properties of PDAC TICs

**Figure 2:**
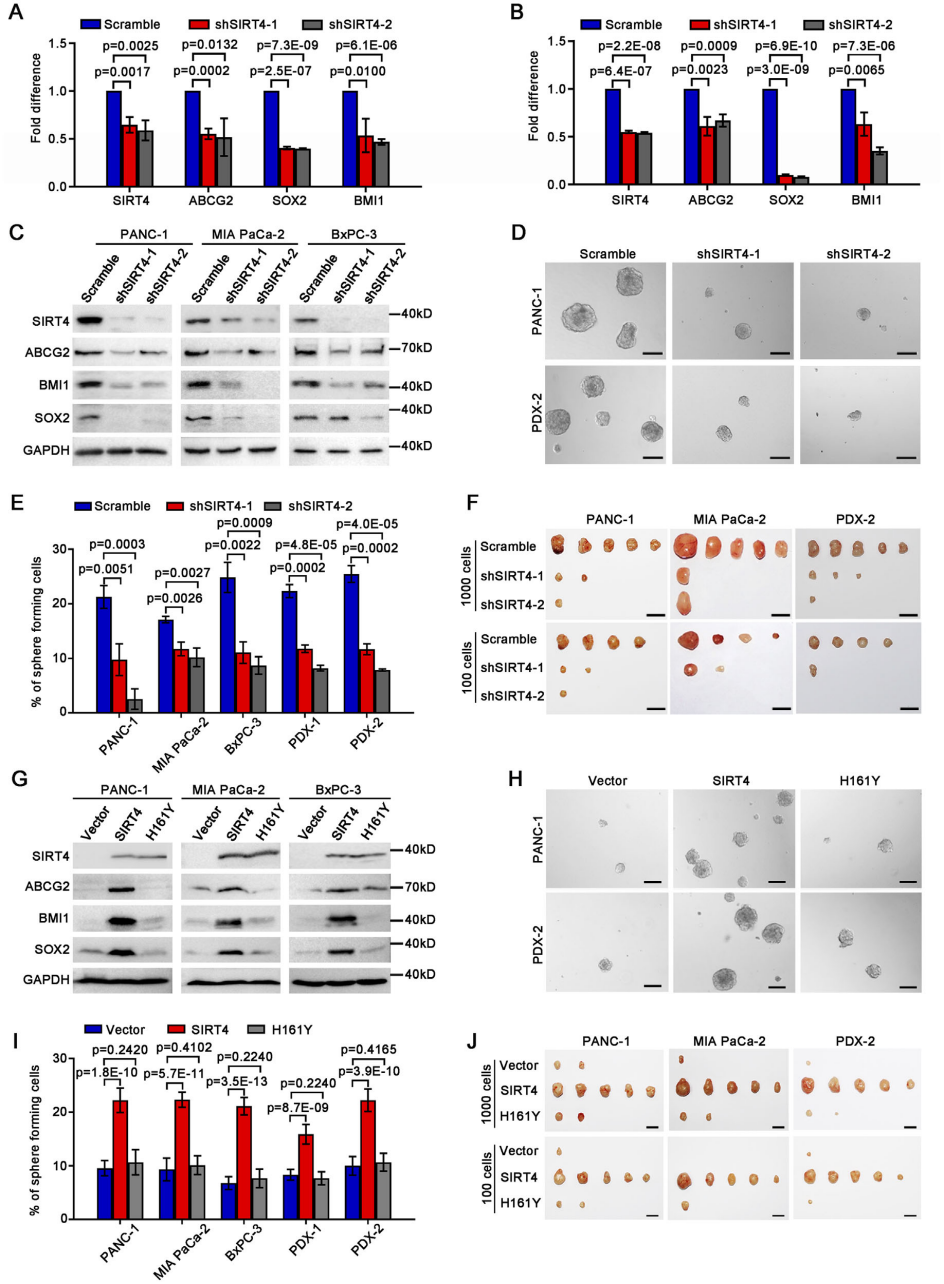
SIRT4 promotes the stem cell‐like properties of PDAC. A,B) Quantitative RT‐PCR analysis of the expression of the indicated genes in α2δ1^+^ PANC‐1 cells (A) and MIA PaCa‐2 cells (B) after *SIRT4* knockdown with its specific shRNAs (*n* = 3). β‐actin served as an internal reference. C) Western blot analysis of the expression of the indicated molecules in purified α2δ1^+^ subsets from the indicated sources after *SIRT4* knockdown with shRNAs. D) Representative phase contrast micrographs showing the spheroids formed by α2δ1^+^ cells sorted from the indicated sources following knockdown the expression of *SIRT4* by specific shRNAs. Scale bars, 100 µm. E) Histograms showing the effect of *SIRT4* knockdown on the spheroid formation capacity of FACS‐sorted α2δ1^+^ subsets from the indicated sources. Cells were seeded in 96‐well plates at 100 cells per well (*n* = 6). F) Photographs showing the dissected tumors formed by the indicated α2δ1^+^ cells infected with the indicated lentiviruses. Scale bars, 1 cm. G) Western blot analysis of the expression of the indicated molecules in FACS‐purified α2δ1^¯^ cells overexpressing the indicated constructs. H) Representative phase contrast micrographs showing the spheroids formed by α2δ1^¯^ cells overexpressing the indicated constructs. Scale bars, 100 µm. I) Histograms showing the spheroid formation efficiencies in the indicated α2δ1^¯^ cells overexpressing the indicated constructs. Cells were plated at 100 cells per well in 96‐well plates (*n* = 3). J) Photographs showing the dissected tumors formed in NOD/SCID mice injected s.c. with the indicated α2δ1^¯^ cells overexpressing the indicated constructs. Scale bars, 1 cm. Data in A, B, E, and I were the mean ± SD of three independent experiments. Unpaired two‐tailed Student's *t*‐test was used for statistical analysis.

To address the functional significance of SIRT4 expression in α2δ1^+^ PDAC TICs, we knocked down the expression of *SIRT4* in the α2δ1^+^ TICs purified from the PDAC cell lines PANC‐1, MIA PaCa‐2 and BxPC‐3 cells using shRNAs against *SIRT4*. The knockdown of *SIRT4* in these α2δ1^+^ cells led to significant downregulation of the expression of a panel of stemness‐associated genes including *ABCG2*, *BMI1*, and *SOX2*, compared with the control cells infected with lentivirus harboring scramble shRNA (**Figure** **2**A–C). Furthermore, the spheroid formation abilities of these α2δ1^+^ fractions were suppressed remarkably following the knockdown of SIRT4 by shRNAs (Figure 2D,E; Figure S1A, Supporting Information). These cells were then subcutaneously transplanted into NOD/SCID mice, at serial dilutions of 1000 and 100 cells, to evaluate their tumorigenic potential. The tumorigenicity of the α2δ1^+^ subpopulations was dramatically retarded following SIRT4 knockdown, as evidenced by the decrease in both the TIC frequencies and formed tumor sizes (Figure 2F; Figure S1B and Table S1, Supporting Information). The effects of SIRT4 knockdown on the spheroid formation and tumorigenicity of α2δ1^+^ TICs were finally validated using two PDAC PDX models. As expected, the spheroid formation abilities and tumorigenicity of the α2δ1^+^ TICs sorted from two PDX models were also inhibited significantly after the knockdown of SIRT4 expression (Figure 2D–F; Figure S1A,B and Table S1, Supporting Information). These data indicate that SIRT4 is required for the maintenance of the stem cell‐like properties of α2δ1^+^ PDAC TICs.

### 2.3 SIRT4 Drives the Stem Cell‐Like Properties of PDAC TICs via Its Enzymatic Activity

To test whether SIRT4 plays any role in the acquisition of stem cell‐like properties, we ectopically expressed SIRT4 in the α2δ1^¯^ subpopulation sorted from the PDAC cell lines PANC‐1, MIA PaCa‐2, and BxPC‐3 by infecting with lentivirus harboring SIRT4 expression cassette. Forced expression of SIRT4 resulted in the upregulation of stem cell‐related genes detected, including *ABCG2*, *BMI1*, and *SOX2* (Figure 2G). Moreover, the spheroid formation abilities of these subpopulations increased significantly after forced expression of *SIRT4* (Figure 2H,I; Figure S1C, Supporting Information). We next transplanted these cells into NOD/SCID mice to assay their tumorigenicity at serial dilutions of 1000 and 100 cells. Overexpression of *SIRT4* led to enhanced tumorigenicity of the α2δ1^¯^ PANC‐1 and MIA PaCa‐2 cells, as evidenced that the cells overexpressing *SIRT4* generated tumors in all the transplanted mice, whereas the control cells only formed 1–2 much smaller nodules for each group (Figure 2J; Table S2, Supporting Information). The promoting roles of *SIRT4* in the spheroid formation abilities and tumorigenic potential were also confirmed by forced expression of *SIRT4* in the α2δ1^¯^ subsets purified from two PDX models (Figure 2H–J; Figure S1C,D, Supporting Information).

The 161^st^ histidine (H) of SIRT4 has been identified as a critical site for its catalytic enzyme activity, and the mutation of H to Tyrosine (Y) at this site resulted in the loss of its enzymatic activity.^[^^12]^ Hence, we constructed this catalytically inactive mutant of SIRT4 (SIRT4‐H161Y), and ectopically expressed it in the α2δ1^¯^ PANC‐1 and MIA PaCa‐2 cells to address whether the promoting role of *SIRT4* on the stem cell‐like properties of PDAC TICs, were dependent on its enzymatic activity. As shown in Figure 2G–J, SIRT4‐H161Y failed to induce the upregulation of stemness‐associated genes detected, including ABCG2, BMI1 and SOX2. The spheroid formation abilities and tumorigenicity of these cells overexpressing SIRT4‐H161Y were indistinguishable from the vector alone control cells. The inability of SIRT4‐H161Y to promote stem cell‐like properties was further validated using α2δ1^¯^ cells purified from the 2 PDX models (Figure 2H–J; Figure S1C,D, Supporting Information).

All the above data demonstrate that SIRT4 induces the stem cell‐like properties of PDAC cells, which is dependent on its enzymatic activity.

### 2.4 SIRT4 Leads to ENO1 Deacetylation at Multiple Lysine Residues

**Figure 3:**
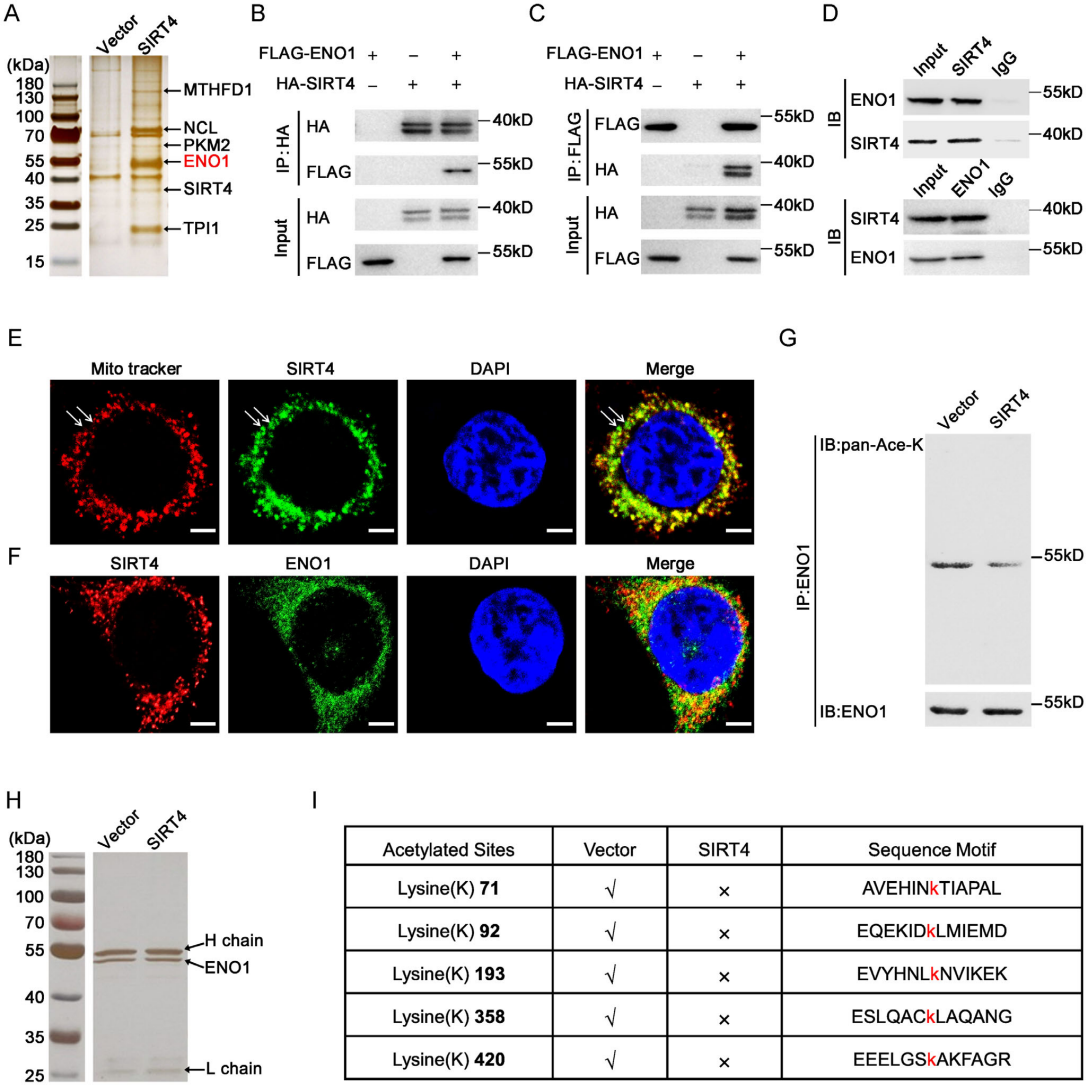
SIRT4 interacts with ENO1 in PDAC cells. A) SDS‐PAGE analysis of the immunoprecipitated products with FLAG‐resin in PANC‐1 cells overexpressing SIRT4‐FLAG construct. Precipitated products in the cells transfected with vector alone serve as a control. Representative candidates of each band identified by mass spectrum are shown. B,C) Western blot analysis with the indicated antibodies of the immunoprecipitation products by the antibody against HA (B) or FLAG (C) from HEK293T cells transfected with HA‐tagged SIRT4 and FLAG‐tagged ENO1 constructs. D) Western blot analysis of the immunoprecipitated products by the indicated antibodies in the PANC‐1 cell line. E) Confocal micrographs showing the subcellular location of SIRT4 protein in PANC‐1 cells. Mito tracker was used to visualize mitochondria and nuclei were stained with DAPI. The white arrows indicating SIRT4 outside the mitochondria. Scale bars, 5 µm. F) Confocal images showing the subcellular location of ENO1 and SIRT4 protein in PANC‐1 cells. DAPI was used to stain nuclei. Scale bars, 5 µm. G) Western blot results demonstrating the change of the acetylated level of the immunoprecipitated ENO1 in α2δ1^¯^ PANC‐1 cells after SIRT4 overexpression. H) ENO1 was immunoprecipitated from the cell lysates of α2δ1^¯^ PANC‐1 cells infected with vector alone or SIRT4‐overexpressing lentivirus and was separated by SDS‐PAGE for acetylation analysis by mass spectrum. I) The list of the acetylated lysine sites of ENO1 in the α2δ1^¯^ PANC‐1 cells infected with the lentivirus harboring empty vector or SIRT4‐expressing cassette identified by mass spectrometry.

To understand the molecular mechanism(s) underlying the roles of *SIRT4* in driving the stem cell‐like properties of PDAC TICs, we performed immunoprecipitation assay using anti‐FLAG M2 beads in PANC‐1 cells ectopically expressing *SIRT4*‐FLAG or the empty vector. Compared with the control, the anti‐FLAG M2 beads incubated with the *SIRT4 *‐FLAG cell lysates precipitated SIRT4 itself, as expected, and several other top candidates including ENO1 as revealed by mass spectrometry analysis of the bands specifically precipitated from the *SIRT4 *‐FLAG cell lysates (**Figure** **3**A). We chose ENO1 for further characterization because of its critical role in glycolysis and cancer. Western blot results confirmed that SIRT4 (probed with anti‐HA antibody) presented specifically in the anti‐FLAG M2 precipitated product of the cell lysate of 293FT cells co‐transfected with SIRT4‐HA and ENO1‐FLAG constructs, while ENO1 (probed with anti‐FLAG antibody) could also be precipitated from the 293FT cells co‐transfected with the two constructs using anti‐HA antibody (Figure 3B,C). We next performed bidirectional co‐immunoprecipitation using extracts of PANC‐1 cells with antibodies against SIRT4 and ENO1 to verify the physiological binding between SIRT4 and ENO1. As shown in Figure 3D, ENO1 was co‐immunoprecipitated by antibody against SIRT4, and vice versa, demonstrating that the two proteins bind each other specifically at endogenous levels. Immunofluorescent staining of SIRT4 and ENO1, together with a mitochondrial tracker, revealed that the majority of SIRT4 localized in mitochondria, while ENO1 was mainly found in cytoplasm. However, colocalization of SIRT4 and ENO1 was indeed found in some sites, suggesting their spatial interaction (Figures 3E,F).

The fact that SIRT4 and ENO1 interacted each other promoted us to test whether ENO1 was a substrate for the deacetylase activity of SIRT4. Western blot analysis of ENO1 precipitated from the vector alone control and SIRT4‐OE PANC‐1 cells with a pan anti‐acetylated lysine antibody showed that the total acetylated lysine of ENO1 reduced significantly in the SIRT4‐OE cells, compared with the control ones (Figure 3G). Further analysis of the acetylation status of the immunoprecipitated ENO1 using high performance liquid chromatography/mass spectrometry (HPLC/MS) identified a number of acetylated lysine residues, including K71, K92, K193, K358 and K420, that were exclusively presented in the ENO1 immunoprecipitated from the control cells, but not in the SIRT4‐OE PANC‐1 cells (Figure 3H,I; Figure S2, Supporting Information).

Taken together, these data demonstrate that SIRT4 might serve as a deacetylase that deacetylates ENO1 at multiple candidate lysine residues.

### 2.5 Deacetylated ENO1 at K358 Promotes the Stem‐like Properties of PDAC TICs

**Figure 4:**
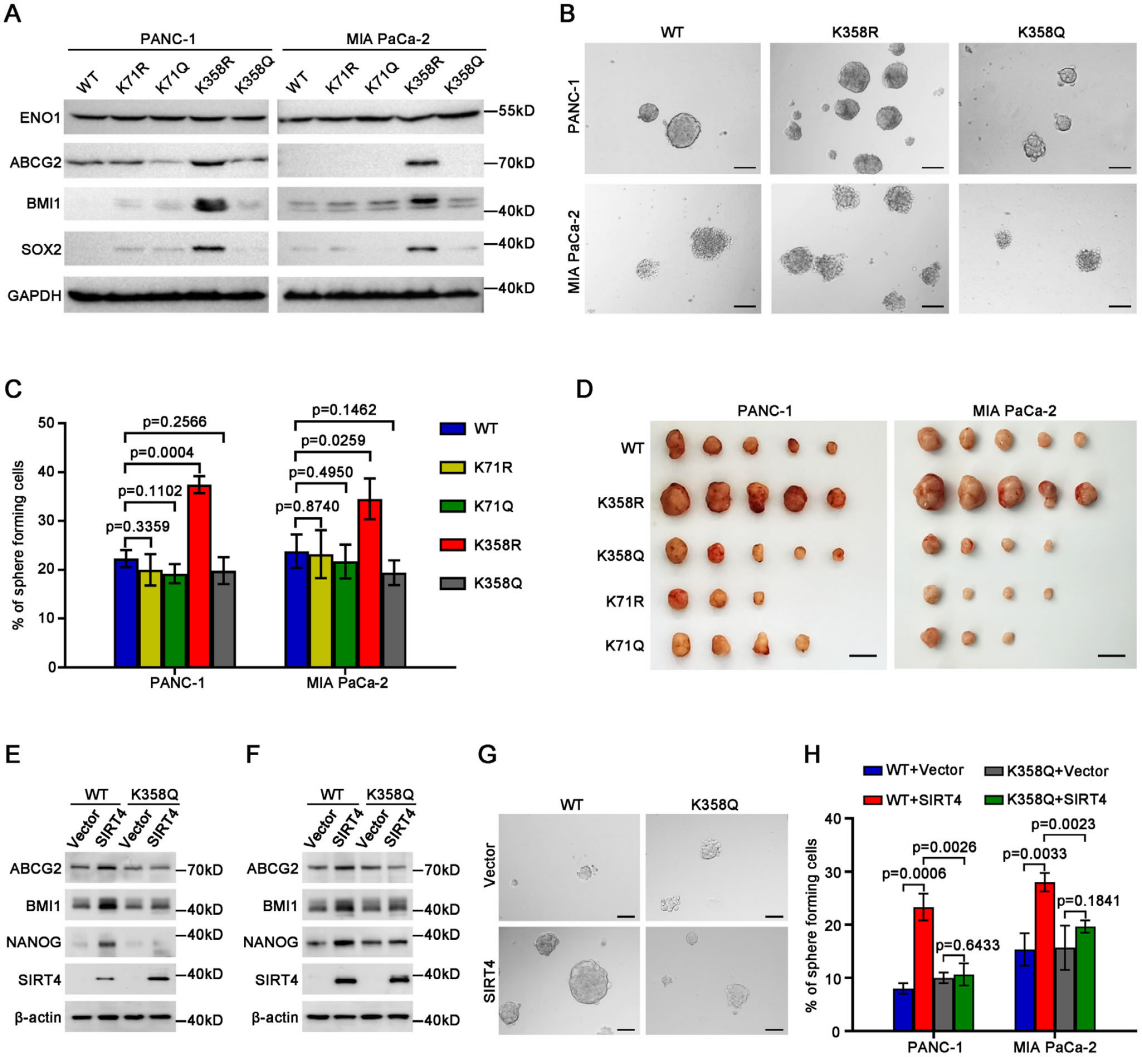
The deacetylation of ENO1 at K358 promotes stem cell‐like traits in PDAC cells. A) Western blot results showing the expression of the indicated molecules in the ENO1‐knockout (ENO1‐KO) cell lines expressing the indicated sgRNA‐resistant constructs. B) Representative images demonstrating the spheroid formed by the indicated cells. Scale bars, 100 µm. C) Histograms showing the spheroid formation efficiencies of the cells expressing each of the indicated ENO1 mutants. Cells were plated at 100 cells/well in 96‐well plates (*n* = 6). D) Photograph demonstrating the dissected tumors formed in NOD/SCID mice by transplanting 1000 cells of the indicated cells at each site. Scale bars, 1 cm. E,F) Western blot results showing the expression of the indicated molecules in the ENO1‐KO PANC‐1 (E) and MIA PaCa‐2 (F) cells expressing wild‐type ENO1(WT) or ENO1^K358Q^ (K358Q) mutant after forced expression of *SIRT4*. G) Representative phase contrast micrographs demonstrating the spheres formed by the ENO1‐KO PANC‐1 cells expressing wild‐type ENO1(WT) or ENO1^K358Q^ (K358Q) mutant after forced expression of *SIRT4*. Cells were seeded at 100 cells/well in 96‐well plates (*n* = 6). Scale Bars, 50 µm. H) Histograms showing the spheroid formation efficiencies of the indicated ENO1‐KO cells expressing wild‐type ENO1(WT) or ENO1^K358Q^ (K358Q) mutant after forced expression of **SIRT4**. Data in (C) and (H) are presented as mean ± SD of three independent experiments. Unpaired two‐tailed Student's *t*‐test was used for statistical analysis.

To determine which deacetylated lysine residue(s) of ENO1 was responsible for the acquisition of stem cell‐like properties of PDAC TICs, we first reconstituted the expression of sgRNA‐resistant wild type (WT), nonacetylatable mutants K71R, K92R, K193R, K358R, and K420R, individually, in PANC‐1 and MIA PaCa‐2 cells with endogenous ENO1 knockout (ENO1‐KO) by sgRNA – guided CRISPR/Cas9 gene editing (Figure S3A, Supporting Information). Initial screening using spheroid formation assay identified that the cells expressing K358R had the highest sphere formation efficiency among the wild type (WT) and mutant ENO1‐OE cells (Figure S3B,C, Supporting Information). We therefore focused on K358 for further characterization with K71 and WT as controls. Additional sgRNA‐resistant mutants that replaced K71 and K358 with acetyl‐mimetic glutamine (Q), i.e., K71Q and K358Q expression vectors, were constructed. These constructs were expressed in ENO1‐KO PANC‐1 and MIA PaCa‐2 cells by lentivirus infection to assay their stem cell‐like properties. Compared with the cells expressing other constructs, those cells expressing K358R expressed the highest levels of stemness‐related molecules including ABCG2, BMI1, and SOX2 (**Figure** **4**A), initiated the growth of spheres with the highest efficiencies (Figure 4B,C; Figure S3D, Supporting Information), and generated tumors with the largest sizes (Figure 4D; Table S3, Supporting Information).

Moreover, forced expression of SIRT4 in the ENO1‐KO PANC‐1 and MIA PaCa‐2 cells overexpressing ENO1^K358Q^ failed to induce the upregulation of stemness‐associated genes detected, including *ABCG2*, *BMI1* and *NANOG*, and enhance the spheroid formation efficiencies of these cells, whereas overexpression of SIRT4 in the ENO1‐KO cells overexpressing ENO1^WT^ promoted the expression of stemness‐associated genes and spheroid formation efficiencies significantly (Figure 4E–H; Figure S3E, Supporting Information), suggesting that the deacetylation of ENO1 at K358 is required for the role of SIRT4 in promoting the stemness of PDAC.

Collectively, these data indicate that the deacetylation of ENO1 at K358 is both sufficient and required for the SIRT4‐promoted role in stem cell‐like properties of PDAC.

### 2.6 Confirmation of ENO1 Deacetylation at K358 by SIRT4 in PDAC TICs

**Figure 5:**
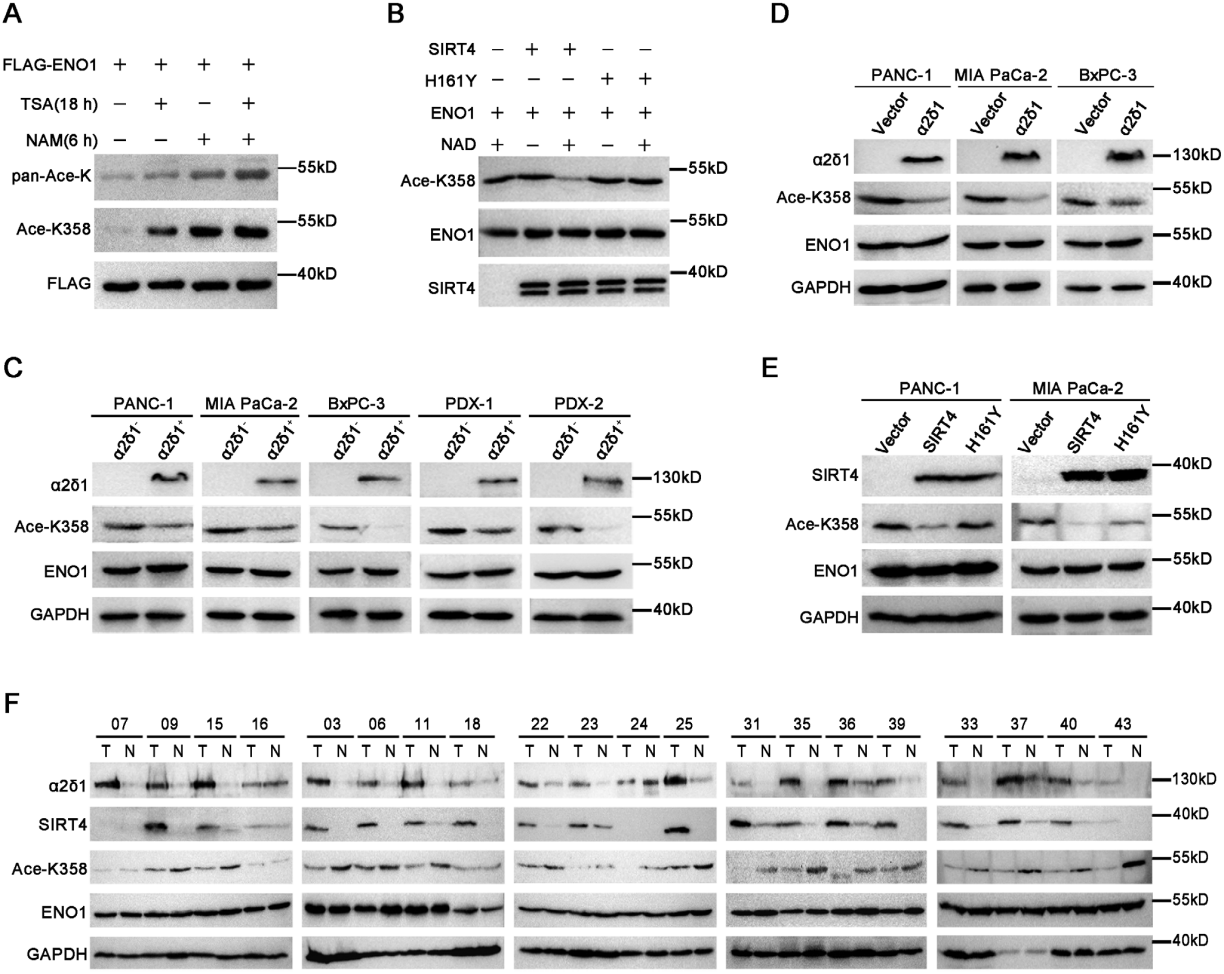
ENO1 is deacetylated at K358 by SIRT4 in PDAC. A) Western blot analysis of the immunoprecipitated FLAG‐ENO1 by FLAG‐resin from HEK293T cells which was transiently transfected with FLAG‐ENO1 construct, followed by the treatment with the indicated reagents. TSA (10 µm), NAM (10 µm). B) In vitro deacetylation assay showing the deacetylation of ENO1 at K358 by SIRT4. The recombinant ENO1 was incubated with purified SIRT4 in the deacetylation assay buffer, and the ENO1 K358 acetylation level was analyzed by Western blotting. C) Western blot results demonstrating the acetylation status of ENO1 at K358 in sorted α2δ1^¯^ and α2δ1^+^ fractions from the indicated sources. D) The acetylation levels of ENO1‐ K358 in the indicated cells overexpressing α2δ1 as detected by Western blot. E) Western blot results showing the acetylation levels of ENO1‐K358 in the indicated α2δ1^¯^ cells overexpressing SIRT4 or mutant SIRT4^H161Y^. F) Western blot results demonstrating the expression of the indicated molecules in 20 pairs of tumor tissues (T) and adjacent normal tissues (N) from PDAC patients.

To further characterize K358 deacetylation, we first generated a site‐specific antibody against acetylated K358 of ENO1(Ace‐K358) (Figure S4A, Supporting Information). It did not react with the cell lysates from ENO1 – KO PANC‐1 and MIA PaCa‐2 cells, while it detected the ENO1 of the cells ectopically expressing ENO1^K358R^ or ENO1^K358Q^ mutants just above background (Figure S4B,C, Supporting Information), confirming the specificity of the antibody. The acetylation of exogenous ENO1‐K358 increased more dominantly after the treatment with nicotinamide (NAM), a deacetylase inhibitor of Sirtuins, compared with the treatment with an inhibitor of histone deacetylases HDAC I and II, trichostatin A (TSA) (**Figure** **5**A), suggesting that the deacetylation of K358 was mainly mediated by Sirtuin family members. Further in vitro deacetylase assay using purified ENO1 and SIRT4 confirmed that SIRT4 was able to deacetylate ENO1‐K358 directly, whereas the deacetylase‐dead mutant SIRT4^H161Y^ led to little change on the acetylation level of K358 (Figure 5B).

We next detected the acetylation levels of ENO1 at K358 in the α2δ1^+^ and α2δ1^¯^ fractions purified from PANC‐1, MIA PaCa‐2 and BxPC‐3 cell lines as well as 2 PDX models by Western blots. The levels of Ace‐K358 were much weaker in the α2δ1^+^ TICs than their respective negative ones, whereas there was no difference found on the total ENO1 levels between both fractions from each source (Figure 5C). Forced expression of α2δ1 or SIRT4 in the pancreatic cancer cell lines led to significant downregulation of Ace‐K358 levels with little effects on the total ENO1 levels (Figure 5D,E), whereas the deacetylase‐dead mutant SIRT4^H161Y^ had no significant effect on the levels of Ace‐K358 compared with the vector alone control cells (Figure 5E).

Finally, we analyzed the expression of α2δ1, SIRT4, and Ace‐ENO1‐K358 in randomly selected 20 pairs of PDAC and adjacent normal tissues by Western blot to validate the relationship between these molecules. As shown in Figure 5F, the expression of SIRT4 was positively correlated with that of α2δ1, which was expressed at much higher levels in the PDAC tissues than in the matched normal tissues, while the expression of Ace‐ENO1‐K358 was downregulated in the PDAC tissues, compared with the matched normal tissues, in 17 cases out of 20 cases detected. The expression levels of Ace‐K358 were negatively correlated with those of SIRT4 and α2δ1.

Altogether, these data confirm that SIRT4 deacetylates ENO1 at K358 directly in PDAC TICs, as a result of α2δ1 expression.

### 2.7 Deacetylated K358 Enhances ENO1 Enzymatic Activity and Promotes Glycolysis

**Figure 6:**
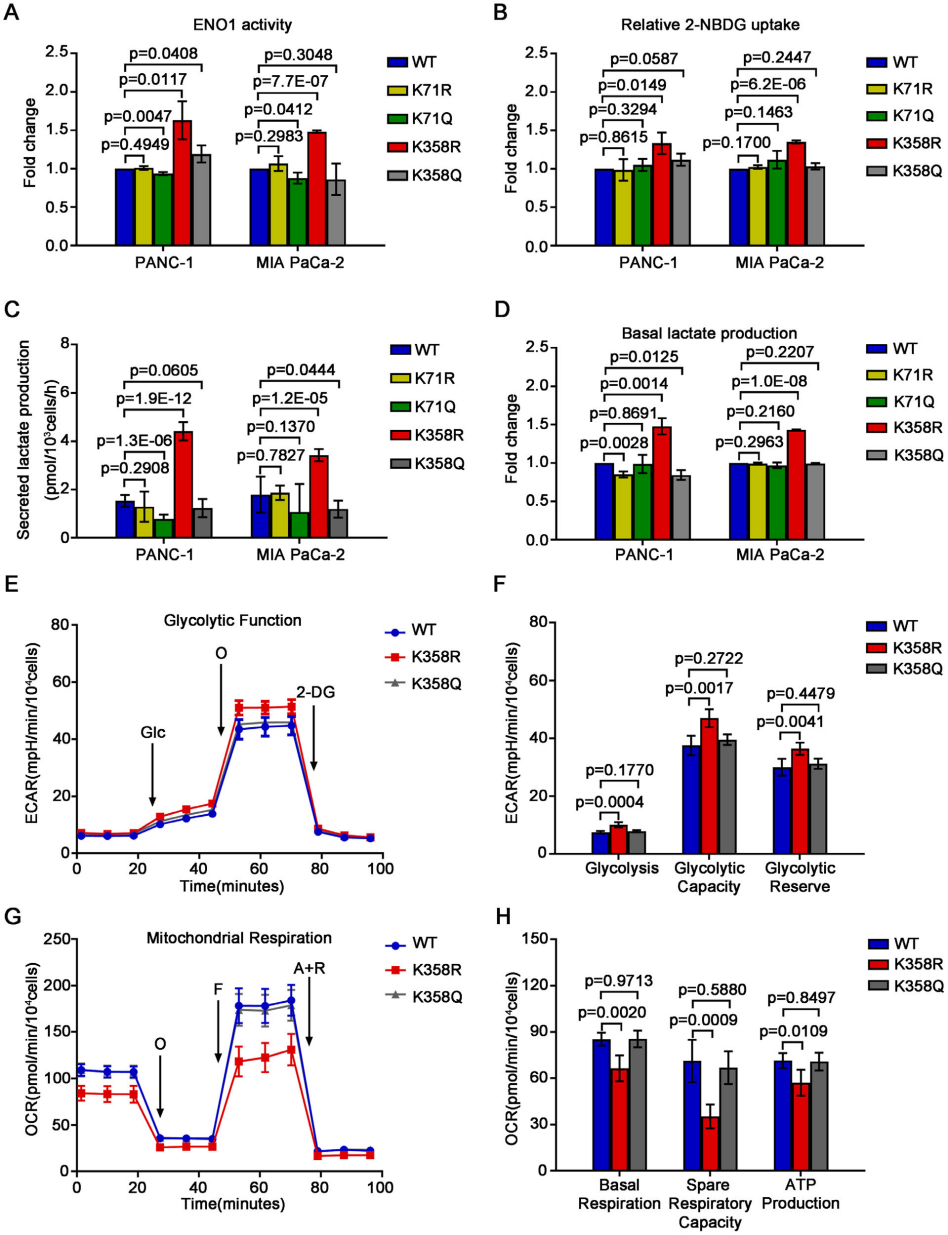
The deacetylation of ENO1‐K358 enhances the enzymatic activity of ENO1 and glycolysis. A) Histograms demonstrating the enzymic activity of ENO1 in the ENO1‐KO cells overexpressing the indicated sgRNA‐resistant constructs (*n* = 3). B) Flow cytometry analysis for glucose uptake capacity using a fluorescence‐labeled glucose analogue, 2‐NBDG. Histograms showing the 2‐NBDG uptake rate of each ENO1 mutant in the indicated ENO1‐KO cells (*n* = 3). C,D) Histograms representing the secreted (C) and intercellular (D) lactate production of the ENO1 mutants in the indicated ENO1‐KO cell line (*n* = 3). E,F) The ECAR curve (E) and histograms (F) exhibiting the changes of glycolysis, glycolytic capacity, and glycolytic reserve in ENO1‐KO PANC‐1 cells overexpressing the indicated constructs (*n* = 5). G,H) The OCR curve (G) and histograms (H) demonstrating the changes in basal respiration, spare respiratory capacity, and ATP production in PANC‐1 cells expressing specific ENO1 mutants (*n* = 5). Abbreviations: A, antimycin A; F, FCCP; Glc, glucose; O, oligomycin; R, rotenone. Data were presented as mean ± SD. Unpaired two‐tailed Student's *t*‐test was used for statistical analysis.

We next measured the enzymatic activity of ENO1 to address whether the deacetylation of K358 has any effect on its catalytic activity. The activity of ENO1 in ENO1‐KO PANC‐1 and MIA PaCa‐2 cells expressing K358R increased by as many as 55.28%, and 48.09%, respectively, compared with that in the cells expressing WT, while there was no significant difference in the ENO1 activity between WT and K71R, K71Q, and K358Q (**Figure** **6**A; Figure S4D–G, Supporting Information). Further direct measurement using purified ENO1 variant proteins expressed in 293FT cells revealed a similar enzymatic activity trend: K358R had the highest enzymatic activity, and all other variants had very similar activities (Figure S4H,I, Supporting Information). These data demonstrate that the deacetylation of ENO1 at K358 enhances its enzyme activity.

Since the major role of ENO1 is participating glycolysis, we then detected whether the deacetylation at K358 of ENO1 has any effects on glucose uptake and lactate production. The uptake of glucose in the ENO1‐KO PANC‐1 and MIA PaCa‐2 cells expressing K358R increased remarkably by about 33.2% and 35.0%, respectively, compared with the respective control cells expressing WT, while no significant difference was observed for the glucose uptake between the WT cells, and the cells expressing K71R, K71Q, or K358Q as detected by 2‐NBDG uptake assay (Figure 6B). Moreover, the lactate secreted into the culture medium by the ENO‐KO PANC‐1 and MIA PaCa‐2 cells ectopically expressing K358R was about 2.87 and 1.91 times that secreted by those cells expressing WT, respectively, the highest levels among the cells expressing WT, or any other mutants (Figure 6C). Consistently, the lactate in the cell lysates of the cells expressing K358R was also the highest among the cells tested (Figure 6D).

We finally performed a cell glycolysis stress test on the ENO1‐KO PANC‐1 cells expressing WT, K358R, or K358Q ENO1 using seahorse assay. Compared with the cells expressing WT, or K358Q, the cells expressing K358R showed enhanced glycolysis, glycolytic capacity, and glycolytic reserve (Figure 6E,F). Further mitochondrial stress test demonstrated that the basal respiration, maximum respiration, and ATP production levels in the K358R expressing PANC‐1 cells were much lower than those in the WT and K358Q cells (Figure 6G,H).

All of these data confirmed that the deacetylation of ENO1 at K358 reprogrammed the mitochondrial oxidative phosphorylation (OXPHOS) metabolism to glycolysis, producing more lactate.

### 2.8 SIRT4 Increases ENO1 Enzymatic Activity and Promotes Glycolysis

**Figure 7:**
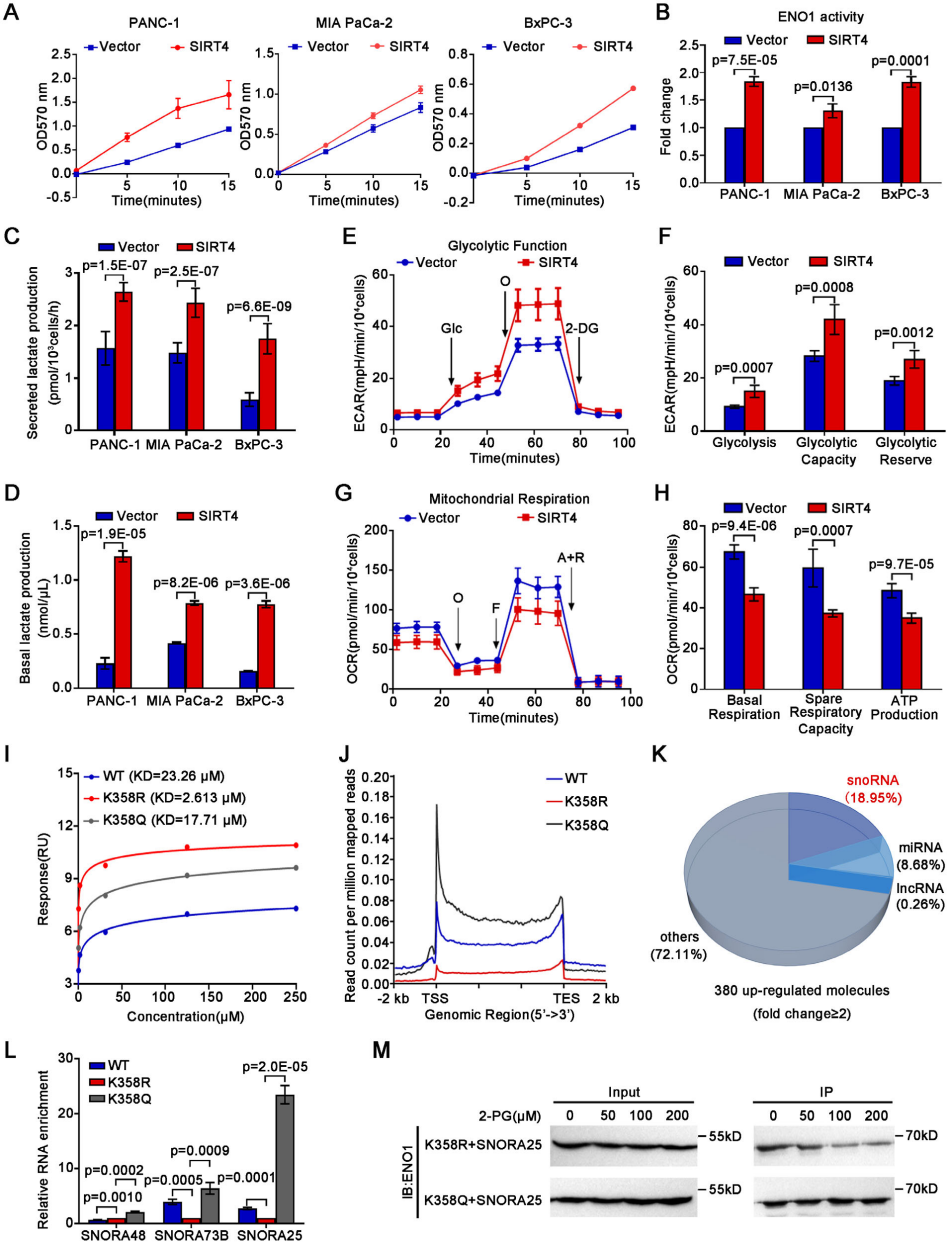
SIRT4 enhances glycolysis and its deacetylation of ENO1‐K358 attenuates the binding of ENO1 to SNORA25. A) Line graphs showing the effects of *SIRT4* on the enzymic activity of ENO1 in the indicated α2δ1^¯^ cells (*n* = 3). B) Histograms showing the fold change of the enzymic activity of ENO1 after forced expression of *SIRT4* in the sorted α2δ1^¯^ cells from the indicated cell lines (*n* = 3). C,D) The secreted (C) and intercellular (D) lactate production were determined in the indicated α2δ1^¯^cells overexpressing *SIRT4* (*n* = 3). E) ECAR curve showing the changes of glycolysis of α2δ1^¯^ PANC‐1 cells after forced expression of *SIRT4* (*n* = 5). F) Histograms showing the changes of glycolysis, glycolytic capacity, and glycolytic reserve following forced expression of *SIRT4* in α2δ1^¯^ PANC‐1 cells (*n* = 5). G) OCR curve showing the change of mitochondrial respiration of α2δ1^¯^ PANC‐1 cells after forced expression of *SIRT4* (*n* = 5). H) Histograms showing the changes in basal respiration, spare respiratory capacity, and ATP production following forced expression of *SIRT4* in α2δ1^¯^ PANC‐1 cells (*n* = 5). I) The steady‐state affinity (binding at equilibrium) of purified wild‐type ENO1, ENO1^K358R^, ENO1^K358Q^ protein with substrate 2‐PG as measured by SPR assay. J) Global RIP‐seq map of bound RNA for wild‐type ENO1, ENO1^K358R^, and ENO1^K358Q^ mutants in the ENO1‐KO PANC‐1 cells expressing the respective sgRNA‐resistant mutants. K) Pie chart showing the distribution proportion of RNAs bound by ENO1^K358Q^ vs ENO1^K358R^, based on the RIP‐seq data in (J). L) Quantitative RT‐PCR results of SNORA48, SNORA73B and SNORA25 after RIP in the ENO1‐KO PANC‐1 cells expressing the respective sgRNA‐resistant mutants (*n* = 3). β‐actin was used as an internal reference. M) Western blot analysis of the immunoprecipitated products by SNORA25 from the mixtures of purified ENO1^K358R^, ENO1^K358Q^ protein with biotinylated labeled SNORA25 and 2‐PG. Data in B, C, D, F, H, and L were presented as mean ± SD. Unpaired two‐tailed Student's *t*‐test was used for statistical analysis.

To validate that the promoting role of deacetylated ENO1 in glycolysis was indeed related to SIRT4, we first measured the enolase activity in SIRT4‐OE PANC‐1, MIA PaCa‐2, and BxPC‐3 cells. In consistent with the results on K358R, the enolase activities increased remarkably by as many as 83.8%, 30.6%, and 83.0% in SIRT4‐OE PANC‐1, MIA PaCa‐2, and BxPC‐3 cells, respectively, over those in the respective vector alone control cells (**Figure** **7**A,B). Furthermore, the glucose uptake in SIRT4‐OE PANC‐1 and MIA PaCa‐2 cells increased by 28.78% and 19.61% over that in the respective control cells, respectively (Figure S5A, Supporting Information). In consistent with increased ENO1 enzymatic activity, forced expression of *SIRT4* in PANC‐1, MIA PaCa‐2, and BxPC‐3 cells led to increased secretion of lactate by 68.46%, 64.21%, and 196.36%, respectively, over the respective control cells (Figure 7C). Moreover, the intracellular lactate levels in SIRT4‐OE PANC‐1, MIA PaCa‐2, and BxPC‐3 cells were about 5.3, 1.9, and 4.9 times of those in the respective control cells, respectively (Figure 7D).

To further test the role of SIRT4 in the regulation of glucose metabolism in PDAC cells, we performed a cell glycolysis stress test in SIRT4‐OE PANC‐1 and MIA PaCa‐2 cells. As shown in the extracellular acidification rate curves, forced expression of SIRT4 resulted in significantly up‐regulated glycolysis, glycolytic capacity, and glycolytic reserve in both cell lines (Figure 7E,F; Figure S5B,C, Supporting Information). Consistently, the relative intracellular abundance of key intermediate metabolites related to glycolysis, such as D‐Glucose, Glucose 6‐P, Fructose 6‐P, PEP, and Pyruvate was all significantly increased in SIRT4 overexpressed PANC‐1 cells compared with the control cells (Figure S5D, Supporting Information). Further cell mitochondrial stress assay revealed that SIRT4 overexpression in PANC‐1 and MIA PaCa‐2 cells led to decreased mitochondrial oxidative phosphorylation levels, as evidenced that the basal respiration, the spare respiratory capacity, and ATP production were decreased significantly after SIRT4 overexpression (Figure 7G,H; Figure S5E,F, Supporting Information), also the intermediates of OXPHOS were widely reduced relative to control α2δ1¯ PANC‐1 cells (Figure S5G, Supporting Information), suggesting the suppression of mitochondrial respiration upon *SIRT4* overexpression in PDAC.

Taken together, these data demonstrated that SIRT4 reprogrammed mitochondrial oxidative phosphorylation to glycolysis with enhanced lactate production to promote the acquirement of TIC properties of PDAC.

### 2.9 Deacetylated ENO1^K358^ Binds Preferentially to Its Glycolytic Substrate 2‐PG rather than to RNAs

To address the molecular basis for the enhanced enzymatic activity of deacetylated ENO1 at K358, we measured the substrate binding capacity of the recombinant ENO1 mutated at K358 with deacetylated mimetic and the acetylated mimetic amino acids using the surface plasmon resonance (SPR) technology. The deacetylated mimetic mutant ENO1‐K358R expressed in *E. Coli* bound to its substrate 2‐PG with a Kd value of 2.613 µM, which was lower than the acetylated mimetic mutant of ENO1 (K358Q, *K*_d_ = 17.71 µm), indicating that the deacetylated ENO1‐K358 had a stronger affinity with its substrate 2‐PG (Figure 7I; Figure S6A–C, Supporting Information) than the acetylated one.

Huppertz et al. reported that the acetylated ENO1 could bind to cellular mRNAs, which competed with its glycolytic substrate 2‐PG, thus inhibiting the catalytic activity of ENO1,^[^ ^45^ ^]^ but it was unclear which lysine(s) in ENO1 was responsible for such riboregulation. We therefore performed RNA binding protein immunoprecipitation and RNA sequencing assay (RIP ‐ seq) to analyze the RNAs precipitated by ENO1 antibody   in PANC‐1 cells infected with lentivirus containing ENO1^WT^, ENO1^K358R^, or ENO1^K358Q^ to test the RNA‐binding characteristics of acetylation/deacetylation of ENO1 at K358. Compared with the WT and the acetylated mimetic mutant ENO1^K358Q^, the deacetylated mimetic mutant ENO1^K358R^ bound to RNAs at a significantly reduced level (Figure 7J). Notably, 72 of the 380 differentially bound RNAs (Fold change≥2) between ENO1^K358Q^ and ENO1^K358R^ were SnoRNAs, accounting for about18.95% of the total differentially bound RNAs in ENO1^K358Q^ (Figure 7K). The RIP‐seq results were further validated by RIP‐qPCR analysis of several selected SnoRNAs including SNORA25, SNORA48, and SNORA73B (Figure S6D, Supporting Information). Consistently, all three SnoRNAs were found binding to ENO1^K358R^ at a much less amount than to the ENO1^K358Q^ (Figure 7L). To address whether the differential binding capacities to SnoRNAs between ENO1^K358Q^ and ENO1^K358R^ affect the enzymatic property of ENO1, we performed competition RIP assay by incubation purified ENO1^K358R^, or ENO1^K358Q^ mutants expressed in *E. Coli* with biotin‐labeled SNORA25 and 2‐PG, the ENO1's catalytic substrate of the forward reaction, followed by immunoprecipitation with Streptavidin Beads 6FF (Smart‐Lifesciences) and Western blotting with anti ‐ ENO1 antibody. While ENO1's substrate 2‐PG competed with SNORA25 for the deacetylated mimetic mutant ENO1^K358R^ binding, the binding of SNORA25 to the acetylated mimetic mutant ENO1^K358Q^ didn't change at all with the addition of 2‐PG (Figure 7M), suggesting that the deacetylation of ENO1 at K358 renders ENO1 with strong binding capacity to its glycolytic substrate 2‐PG, whereas the acetylated ENO1 at K358 stably binds RNAs, mainly SnoRNAs.

Hence, the deacetylation of ENO1‐K358 enables it to be released from RNAs, and preferentially bind to 2‐PG with stronger affinity, enhancing the glycolytic capacity of the cells.

### 2.10 Glycolysis is Responsible for the Role of SIRT4 in Promoting the Stemness

**Figure 8:**
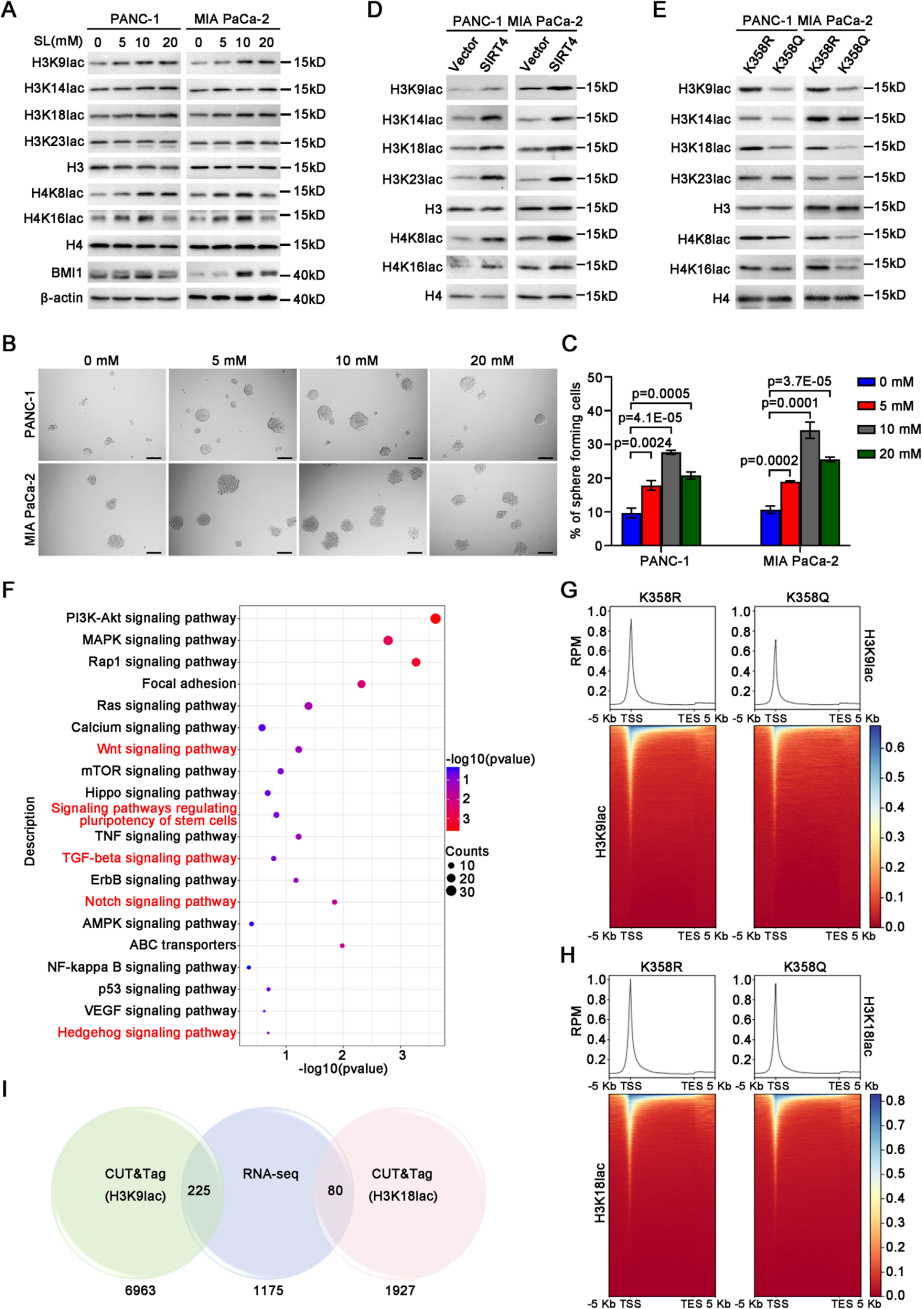
SIRT4‐mediated deacetylation of ENO1‐K358 leads to histone lactylation and epigenetic reprogramming. A) Western blot results showing the lactylation of histones and the expression of the indicated molecules in the indicated cells treated with sodium lactate (SL) for 48 h. B) Representative micrographs showing the spheres formed by the indicated cells treated with sodium lactate. Scale bars, 100 µm. C) Histograms showing the spheroid forming efficiencies of the indicated cells treated with sodium lactate (*n* = 6). Data were the mean ± SD of three independent experiments. Unpaired two‐tailed Student's *t*‐test was used for statistical analysis. D) Immunoblotting results showing the effects of *SIRT4* on the lactylation of histones as detected by Western blot in the indicated cells overexpressing *SIRT4*. E) Western blot results showing the lactylation of histones at the indicated lysine residues in the indicated cells. F) Top pathways (FDR<0.05) identified by KEGG enrichment analysis among the up‐regulated genes in MIA PaCa‐2 cells overexpressing ENO1‐K358R compared with the ENO1‐K358Q ones. G,H) Metaplots and heatmaps showing the occupancy of genome‐wide H3K9lac and H3K18lac binding peaks in a ± 5 kb window surrounding the transcription start sites (TSS) and transcription end sites (TES) in ENO1^K358R^ MIA PaCa‐2 and ENO1^K358Q^ ones. I) Venn diagram showing the overlapped candidate genes upregulated by lactylated H3K9 or H3K18 in CUT&Tag and RNA‐seq data of ENO1^K358R^ MIA PaCa‐2 cells versus ENO1^K358Q^ ones.

The production of lactate as a result of glycolytic switch, has been reported to cause histone lactylation and gene expression,^[^ ^46^, ^47^ ^]^ hence, we treated both the PANC‐1 and MIA PaCa‐2 cells with sodium lactate to mimic the role of lactate to address whether glycolysis is sufficient to promote the stemness and histone lactylation of PDAC. Treatment of these cells with sodium lactate at doses of 5 mm and 10 mm led to histone 3 (H3) and histone 4 (H4) lactylation at multiple sites including H3K9, H3K18, and H4K8, and H4K16 as demonstrated by Western blot with the respective site‐specific anti‐lactylated histone lysine antibodies, although the effect of lactate on the lactylation of histones at some sites (e.g., H4K8, H4K16) was weakened at a dose of 20 mm (**Figure** **8**A). Moreover, the expression of BMI1 and the spheroid formation efficiencies were also increased remarkably following the treatments (Figure 8A–C), suggesting that lactate, the product of glycolysis, itself is sufficient to promote the stemness and histone lactylation of PDAC.

On the contrary, the expression of a panel of stemness‐associated molecules detected, including ABCG2, BMI1, and NANOG, could be suppressed by the treatment of PANC‐1 and MIA PaCa‐2 cells overexpressing SIRT4 with 2‐deoxy‐d‐glucose (2‐DG), an inhibitor of glycolysis, in a dose‐dependent manner (Figure S7A, Supporting Information). Furthermore, the spheroid formation efficiencies of the SIRT4‐OE PANC‐1 and MIA PaCa‐2 cells decreased remarkably after the treatment with 2‐DG also in a dose‐dependent manner (Figure S7B,C, Supporting Information), suggesting that the promoting role of SIRT4 on the stemness was indeed dependent on glycolysis.

### 2.11 SIRT4‐Mediated Deacetylation of ENO1‐K358 Modulates Histone Lactylation and Epigenetic Reprogramming

The aforementioned findings prompted us to hypothesize that SIRT4‐mediated ENO1‐K358 deacetylation drives the TIC properties of PDAC by modulating the pathway(s) involved in stemness regulation via histone lactylation. As demonstrated by Western blotting, forced expression of *SIRT4* in both PANC‐1 and MIA PaCa‐2 cell lines resulted in increased lactylation levels of H3K9, H3K14, H3K18, H4K8, and H4K16 (Figure 8D). Of these lactylated histones, H3K9lac, H3K14lac, and H3K18lac were also consistently upregulated in both the PANC‐1 and MIA PaCa‐2 cell lines expressing ENO1^K358R^, compared with those cells expressing ENO1^K358Q^ (Figure 8E).

Histone lactylation has emerged as a new epigenetic regulator that controls gene expression and cell fate in many systems.^[^ ^46^, ^47^, ^48^ ^]^ Hence, we performed RNA‐seq and genome‐wide Cleavage under Targets and Tagmentation (CUT&Tag) analysis to identify epigenetic reprogramming resulting from SIRT4‐mediated deacetylation of ENO1 at K358 in MIA PaCa‐2 cells. RNA‐seq in MIA PaCa‐2 cells stably expressing ENO1^K358R^ and ENO1^K358Q^ revealed that a total of 1175 genes were up‐regulated and 231 genes were down‐regulated in the cells expressing ENO1^K358R^ compared with those expressing ENO1^K358Q^ (Table S7, Supporting Information). These differentially upregulated transcripts were most enriched in many signal transduction pathways that were essential for stem cell fate determination such as NOTCH, Hedgehog, WNT, and TGF‐β signaling pathways (Figure 8F; Table S8, Supporting Information). In particular, those genes involved in the signaling pathways that regulate pluripotency of stem cells, including *PIK3CD*, *PIK3R3*, *WNT2B*, *WNT10A*, *FGFR4*, *FGFR2*, *FZD4*, *JAK3*, and *MAPK11*, as well as those involved in PD‐L1 expression and PD‐1 checkpoint pathway, such as *CD4*, *MAP2K6*, *MAPK11* were also among the ENO1^K358R^‐upregulated genes (Figure 8F; Table S8, Supporting Information). Further CUT&Tag analysis using the antibodies against lactylated H3K9 and H3K18, the two dominantly upregulated lactylated histones shared by lactate treatment, ectopic expression of *SIRT4*, and ENO1^K358R^, identified 21 937 and 30 411 peaks enriched significantly in MIA PaCa‐2 cells stably expressing ENO1^K358R^ for H3K9lac and H3K18lac, respectively. These binding peaks were highly enriched near transcription starting sites (TSSs), indicating significantly increased H3K9lac and H3K18lac signals within the range of ±5 kb of TSS region in ENO1^K358R^ group relative to ENO1^K358Q^ (Figure 8G,H). Association of these lactylated histone‐bound regions with annotated genes identified 6963 and 1927 H3K9lac‐ and H3K18lac‐bound nearest genes, respectively (Figure 8I; Tables S9 and S10, Supporting Information). Integrating of RNA‐seq with CUT&Tag data identified a total of 225 and 80 overlapped genes with increased mRNA levels and elevated H3K9lac‐, H3K18lac‐bound peaks, respectively, in ENO1^K358R^‐expressing cells (Figure 8I; Tables S11 and S12, Supporting Information), representing directly activated genes by lactylated H3K9 and H3K18, respectively. Notably, some of the genes involved in the aforementioned pathways that regulate stemness, including *BBC3*, *FZD4*, *GLI4*, *HES2*, *HES4*, *MSI1*, *PIK3R3*, *PIK3CD*, and *PTCH2*, were encompassed in the sets (Tables S11 and S12, Supporting Information), suggesting that SIRT4‐mediated deacetylation of ENO1 at K358 to augment the stem cell‐like properties of PDAC via lactate‐mediated histone lactylation and epigenetic reprogramming.

## 3 Discussion

In this study, we identified that the mitochondria Sirtuin family member SIRT4 was transcriptionally upregulated via the α2δ1‐mediated calcium signaling pathway that involved α2δ1‐CaMK2δ‐PKM2‐YAP axis. Furthermore, SIRT4 was functionally sufficient and indispensable for the acquirement and subsequent maintenance of the stem cell‐like properties of α2δ1^+^ TICs of PDAC through deacetylating directly ENO1 at K358, leading to attenuated ENO1's RNA‐binding capacity, enhanced glycolytic substrate 2‐PG affinity, and subsequently robust catalytic activity with boosted glycolytic ability and increased production of lactate. The lactate was able to induce histone lactylation to epigenetically regulate a plethora of signaling pathways that were essential for stemness. Hence, our study reveals an oncogenic role of SIRT4 in promoting stem cell‐like properties and glycolysis, and has linked α2δ1‐mediated calcium signaling to SIRT4‐controlled glycolysis and epigenetic reprogramming involved histone lactylation, uncovering a novel signaling pathway in the determination of the stem cell‐like properties of PDAC TICs.

As a mitochondria Sirtuin, SIRT4 has been demonstrated to play important roles in the controlling of many metabolic pathways such as those involved in glutamine,^[^ ^49^ ^]^ leucine,^[^ ^12^ ^]^ and lipid metabolism^[^ ^18^ ^]^ by regulating multiple mitochondria enzymes. Our finding here that SIRT4 served as a switch between mitochondrial oxidative phosphorylation to glycolysis by deacetylating ENO1 to increase the production of lactate does not exclude other metabolic pathways that might be also involved in the promoting role of SIRT4 in the acquisition of stem cell‐like properties. For example, the increase of acetyl coenzyme A (acetyl‐CoA), a central metabolic intermediate of multiple pathways including branched‐chain amino acid (BCAA) catabolism, has been linked to the tumorigenesis and stem cell‐like properties of a variety of cancers,^[^ ^50^ ^]^ and the level of acetyl‐CoA was indeed elevated significantly in both PANC‐1 and MIA PaCa‐2 cells after forced expression of *SIRT4* (our unpublished data). Nevertheless, our observation that SIRT4 failed to induce stem cell‐like properties in the cells expressing the acetyl‐mimetic mutant ENO1‐K358Q confirms the essential role of the deacetylated ENO1‐mediated lactate production in the acquisition of the stem cell‐like properties of PDAC TICs.

Except for its role in glycolysis, ENO1 could function as an RNA‐binding protein that bound various RNAs such as cellular mRNAs and circular RNAs, modulating their instability and/or itself glycolytic enzyme activity to play important roles in embryonic stem cell differentiation, ferroptosis, and hepatocellular carcinoma metastasis.^[^ ^45^, ^51^, ^52^ ^]^ Huppertz et al. reported that acetylated ENO1 bound RNAs which compete with its substrate 2‐PG, led to suppressed enzymatic activity and glycolysis, thus was associated with embryonic differentiation.^[45^^]^ Our study identified that the acetylation of ENO1 at K358 bound many SnoRNAs including SNORA25, which was not affected by 2‐PG at all. This finding is different from that by Huppertz et al. One possibility is that different acetylation sites in ENO1 might affect the affinity between ENO1 and RNAs or 2‐PG. Future work is required to characterize the structural basis underlying these phenomena. Nevertheless, we propose that acetylation status of ENO1‐K358 serves as a switch between RNA binding and glycolytic substrate 2‐PG binding, thus controlling the glycolytic activity and stem cell‐like properties of PDAC TICs. SIRT4 deacetylates ENO1 at K358, thus serving as an “ignitor” of these processes. We should add that, although ENO1 is not a rate‐limiting enzyme of glycolysis, it might regulate the expression levels or/and the activities of those rate‐limiting enzymes such as HK2 and PKM2 as reported in the literature^[^ ^53^, ^54^ ^]^ via a mechanism to be defined to increase the glycolysis level. In fact, high expression and/or enhanced enzymatic activities of the glycolytic rate‐limiting enzymes such as PKM2 have been observed in many cancers including PDAC.^[^ ^55^, ^56^ ^]^

The glycolysis product lactate could provide carbons to the acetyl‐residues of histones, resulting in lactylated modification of histones to epigenetically reprogram many cellular processes such as gene expression, cell fate determination, cell proliferation, and differentiation, as well as pathological processes including carcinogenesis, metastasis, and treatment resistance.^[^ ^46^, ^57^, ^58^ ^]^ Here, we identified that lactate resulted from SIRT4‐mediated glycolysis led to histone lactylation mainly at H3K9 and H3K18 and acquired stem cell‐like properties of PDAC TICs. The lactylated histones activated directly not only the genes involved in stemness regulation, but also those involved in PD‐L1 expression and PD‐1 checkpoint pathways, suggesting that SIRT4‐mediated histone lactylation play critical roles both in the determination of stem cell‐like properties and the immune evasion of PDAC TICs. However, stem cell‐associated genes such as *ABCG2*, *BMI1*, and *SOX2* were not found among those genes directly regulated by lactylated histones. One possibility is that they might be regulated by the signaling pathways such as NOTCH, Hedgehog, WNT, TGF‐β, and/or Hippo, which were activated by the lactylated histones. Future work is required to define the detailed mechanism involved in the upregulation of these stem cell‐associated genes mediated by SIRT4.

As a second messenger, Ca^2+^ relays signals from various stimulations through several downstream sensor and adaptor proteins including calmodulin (CaM), which subsequently binds and activates downstream enzymes such as Ca^2+^/CaM‐dependent protein kinase II (CaMKII), to control a variety of cellular processes including gene transcription, cell metabolism, proliferation, motility, cell death, and survival.^[^ ^59^ ^]^ It is not surprising that Ca^2+^/CaMKII has been regarded as a central regulator of many physiological activities such as neuronal plasticity and cognitive functions.^[^ ^60^, ^61^ ^]^ Breakdown of Ca^2+^ homeostasis and/or aberrant expression of calcium signaling pathway molecules have been linked to many pathologic processes including cancer.^[^ ^61^, ^62^, ^63^ ^]^ It would be interesting to determine if the calcium‐CaMKIIδ‐SIRT4‐ENO1‐glycolysis‐histone lactylation pathway identified in this study is also present in these calcium‐related physiological and/or pathological processes. Moreover, lactate has been demonstrated as a critical immune modulator for many immune cells,^[^ ^64^, ^65^ ^]^ more work is warranted to address if this signaling plays any role in microenvironment remodeling, and/or the function of immune cells. Finally, the molecules involved in this signaling pathway might be potential targets for therapeutic intervention, as well as prognosis markers, given that the stemness of TICs is associated with treatment resistance and recurrence of cancers.

In summary, our study uncovers novel roles of SIRT4 in controlling glycolysis, histone lactylation, and prompting the stem cell‐like properties of PDAC. Future study is warranted to address whether targeting the CaMKIIδ‐SIRT4‐ENO1‐glycolysis‐histone lactylation pathway could have a therapeutic effect in PDAC by elimination of TICs. Nevertheless, these findings contribute to our understanding of the mechanisms underlying the acquisition and subsequent maintenance of the stem cell‐like properties of PDAC TICs, and provide potential targets for the development of therapeutic strategies of PDAC.

## 4 Experimental Section

### Cell Lines and Clinical Samples

The human pancreatic cancer cell lines PANC‐1, MIA PaCa‐2, and BxPC‐3 were obtained from the American Type Culture Collection (ATCC, Manassas, VA, USA). Cell line authentication was performed using DNA fingerprinting through short tandem repeat (STR) profiling. PANC‐1 and MIA PaCa‐2 cells were cultured in Dulbecco's Modified Eagle's Medium (DMEM), while BxPC‐3 cells were maintained in Roswell Park Memorial Institute (RPMI)‐1640 medium. Both media were supplemented with 10% fetal bovine serum (FBS), 100 U mL^−1^ penicillin, and 100 µg mL^−1^ streptomycin (Invitrogen, Grand Island, NY, USA). Cells were incubated at 37 °C in a humidified atmosphere containing 5% CO_2_.

Patient‐derived xenograft (PDX) models were established as previously described by Liu Ma et al.^[42^^]^ Primary PDAC tissues and matched adjacent non‐tumor tissues were collected from patients who underwent complete surgical resection (R0) at the Department of General Surgery, Peking University Third Hospital.

### Vector Construction and Stable Cell Line Establishment

The construction of α2δ1 overexpression or α2δ1/CaMKIIδ shRNA lentiviral vectors, lentivirus packaging, and cell infection procedures were performed as described in a previous study.^[^ ^42^ ^]^ The coding sequence (CDS) of the human *SIRT4* gene, flanked by BamHI and XhoI restriction sites at the 5′ and 3′ ends, respectively, was synthesized by Sangon Biotech Co., Ltd. (Shanghai, China) and subsequently cloned into the lentiviral shuttle vector plenti6 (Invitrogen, Carlsbad, CA, USA). To knock down *SIRT4* in sorted α2δ1^+^ pancreatic cancer cells, short hairpin RNAs (shRNAs) targeting *SIRT4* were designed and cloned into the lentiviral shuttle vector pSIH‐H1‐Puro. The shRNA sequences used for SIRT4 knockdown are listed in Table S4 (Supporting Information).

Additionally, single guide RNAs (sgRNAs) targeting the ENO1 CDS region were designed and cloned into the lentiCRISPRv2 vector to knockout endogenous ENO1 expression in pancreatic cancer cells, as previously described.^[^^53]^ To generate ENO1 site‐specific mutants, overlap extension PCR was performed to amplify the mutant ENO1 sequences, which were then cloned into the PEE12.4‐DDK expression vector or the PLVX lentiviral vector. Lentiviral constructs were transfected into 293FT cells using the ViraPower Packaging Mix (Invitrogen, Carlsbad, CA, USA) to produce lentiviral particles. Stable cell lines were established through selection with 5 µg mL^−1^ blasticidin, 2 µg mL^−1^ puromycin, or 500 µg mL^−1^ geneticin.

### Protein Extraction and Western Blot

Proteins were extracted using Laemmli sample loading buffer (50 mm Tris‐HCl, pH 6.8, 2% SDS, 10% glycerol, 10% 2‐mercaptoethanol). Total histone proteins were isolated from PDAC cells using a Histone Extraction Kit (Proteintech, Chicago, IL, USA) according to the manufacturer's instructions. Protein samples were separated by sodium dodecyl sulfate‐polyacrylamide gel electrophoresis (SDS‐PAGE) on 10% or 12% gels and then transferred to polyvinylidene fluoride (PVDF) membranes (Millipore, Billerica, MA, USA). Membranes were probed with primary antibodies (Table S5, Supporting Information), followed by incubation with horseradish peroxidase (HRP)‐conjugated goat anti‐rabbit or anti‐mouse secondary antibodies (Jackson ImmunoResearch Laboratories, West Grove, PA, USA). Protein signals were visualized using Immobilon Western Chemiluminescent HRP Substrate (Millipore, Billerica, MA, USA) and detected with a chemiluminescence imager (Minichemi610, Beijing, China).

### Spheroid Formation Assay

To evaluate spheroid formation efficiency, approximately 100 cells per well were seeded into an ultra‐low attachment 96‐well plate (Corning Incorporated Life Sciences, Acton, MA, USA). Cells were cultured in serum‐free DMEM/F12 medium (Gibco, Grand Island, NY, USA) supplemented with epidermal growth factor (EGF, 20 ng mL^−1^; Gibco, Grand Island, NY, USA), basic fibroblast growth factor (bFGF, 20 ng mL^−1^; Gibco, Grand Island, NY, USA), 1× B27 supplement (Invitrogen, Carlsbad, CA, USA), and 2% methylcellulose (Sigma‐Aldrich, St. Louis, MO, USA) at a 1:1 ratio. Cells were incubated at 37 °C in a humidified atmosphere containing 5% CO_2_ for 2–3 weeks. Spheroids larger than 100 µm were photographed and counted using a stereomicroscope (Olympus, Tokyo, Japan).

### In Vivo Tumorigenicity Assay

Cells were suspended in 50 µL of RPMI 1640 or DMEM medium and mixed with an equal volume of Matrigel (BD Biosciences, Bedford, MA, USA). The cell suspension was subcutaneously injected into the backs of 4‐ to 6‐week‐old female NOD/SCID mice (HFK Bioscience Co., Ltd., Beijing, China). Tumor growth was monitored weekly.

### Glucose Uptake Assay

The glucose uptake capacity of PDAC cells was assessed using the fluorescent glucose analog 2‐NBDG (Invitrogen, Carlsbad, CA, USA). Approximately 1 × 10⁶ cells were seeded in 6‐well plates and starved in glucose‐free DMEM for 16–24 h. Cells were then incubated with 50 µM 2‐NBDG at 37 °C for 30 min. After incubation, cells were filtered through a 40‐µm nylon mesh, and fluorescence intensity was measured using a BD Accuri C6 flow cytometer (BD Biosciences, San Jose, CA, USA).

### ECAR and OCR Assays

Metabolic assays were performed using the XF24 analyzer (Agilent Technologies, Santa Clara, CA, USA) according to the manufacturer's instructions. Extracellular acidification rate (ECAR) and oxygen consumption rate (OCR) were measured using the Seahorse XF Glycolysis Stress Test Kit and Seahorse XF Cell Mito Stress Test Kit (Agilent Technologies, Palo Alto, CA, USA), respectively. Briefly, 1 × 10^4^ cells were seeded into a 24‐well XF cell culture microplate and cultured in Seahorse XF DMEM medium for 24 h. The sensor cartridge was hydrated with Seahorse XF Calibrant and incubated overnight at 37 °C in a non‐CO₂ environment. For ECAR measurement, the XF base medium (pH 7.4) was supplemented with 4 mm glutamine, followed by sequential injections of 25 mm glucose, 2 µm oligomycin, and 50 mm 2‐deoxy‐d‐glucose (2‐DG). For OCR measurement, the XF base medium (pH 7.4) was supplemented with 1 mm sodium pyruvate, 4 mm glutamine, and 25 mm glucose, followed by sequential injections of oligomycin (1 µM), carbonyl cyanide‐4‐(trifluoromethoxy)phenylhydrazone (FCCP, 1 µm), and a mixture of antimycin A/rotenone (1 µm each). Data were analyzed using the Seahorse XF Glycolysis/Mito Stress Test Reporter Generator software package.

### Metabolite Extraction and Non‐Targeted Metabolomic Mass Spectrometry Analysis

For metabolite extraction, 2 mL of pre‐chilled 80% methanol (stored at −80 °C) was added to the cell culture plates and incubated at −80 °C for 1 h. Cells were scraped on dry ice and centrifuged at 14 000 × *g* for 20 min at 4 °C. The supernatant containing metabolites was collected and dried using a SpeedVac or lyophilizer, then stored at −80 °C until further analysis. Non‐targeted metabolomic mass spectrometry analysis was conducted at the Metabolomics Facility, Technology Center for Protein Sciences, Tsinghua University (Beijing, China).

### Co‐Immunoprecipitation (Co‐IP)

Cells were lysed in 1% NP‐40 buffer containing 50 mm Tris‐HCl (pH 7.4), 150 mm NaCl, 5 mM EDTA (pH 7.4), and a complete protease inhibitor cocktail (Roche, Mannheim, Germany) at 4 °C for 30 min. The lysates were centrifuged, and the supernatants were incubated with Anti‐FLAG Affinity Gel (Millipore, Billerica, MA, USA) or Anti‐HA Affinity Gel (Sigma‐Aldrich, St. Louis, MO, USA) for 4–6 h at 4 °C. The beads were washed three times with 1% NP‐40 buffer, and bound proteins were eluted using FLAG peptide or by boiling in SDS‐PAGE loading buffer. Eluted proteins were separated by 10% SDS‐PAGE for subsequent Western blot analyis.

For endogenous co‐immunoprecipitation, 5 µg of the indicated antibody or control rabbit/mouse IgG was incubated with Protein A/G Sepharose Fast Flow beads (GE Healthcare, Little Chalfont, Buckinghamshire, UK) for 4–6 h at 4 °C. The antibody‐bead complexes were then added to the pre‐cleared protein lysates and gently rotated overnight at 4 °C. The immunoprecipitates were washed, boiled in SDS‐PAGE loading buffer, and separated by SDS‐PAGE for subsequent immunoblot analysis as described above.

### Silver Staining for Mass Spectrometry

Proteins were separated by 10% SDS‐PAGE and visualized using the Pierce Silver Stain for Mass Spectrometry Kit (Thermo Fisher Scientific, Waltham, MA, USA) according to the manufacturer's instructions.

### Flow Cytometry

Cells were dissociated and resuspended in sterile PBS. For staining, cells were incubated with a monoclonal mouse anti‐α2δ1 antibody (isoform 5, mAb 1B50‐1) pre‐conjugated with fluorescein isothiocyanate (FITC) using a FITC Conjugation Kit (Abcam, Cambridge, UK). An isotype‐matched mouse IgG3 antibody was used as a negative control. After staining, cells were washed twice with sterile PBS and sorted using a FACSAria II flow cytometer (BD Biosciences, San Jose, CA, USA).

### Enolase 1 Activity Assay

To measure the enzymatic activity of ENO1, cell lysates of cultured cells or purified proteins expressed in 293FT cells were analyzed using an Enolase Activity Colorimetric Assay Kit (Abcam, Cambridge, MA, USA) according to the manufacturer's instructions. In brief, 10 µL of each sample was added per well, and the final volume was adjusted to 50 µL with Enolase Assay Buffer. Then, 50 µL of Reaction Mix was added to each well, and the mixture was incubated with gentle shaking at room temperature for 20‐60 min. Absorbance was measured at 570 nm, and enolase activity was calculated based on the H₂O₂ standard curve.

### Lactate Assay

Basal lactate production was quantified using a Lactate Assay Kit (Sigma–Aldrich, St. Louis, MO, USA) following the manufacturer's protocol. Secreted lactate levels in the cell culture supernatant were measured using a Silman M900 Biochemical Process Analyzer (Shenzhen Siemantec Technology Co., Ltd., Shenzhen, China).

### In Vitro ENO1 Deacetylation Assay

Each of FLAG‐SIRT4‐WT, FLAG‐SIRT4‐H161Y, and FLAG‐ENO1 was overexpressed in HEK293FT cells by transfection, and was purified using Anti‐FLAG Affinity Gel. For FLAG ‐ ENO1 purification, HEK293FT cells were treated with the deacetylase inhibitor nicotinamide (10 µM) for 6 hours before the cells were collected. Recombinant ENO1 (0.5 mg mL^−1^) were incubated with purified SIRT4‐WT or SIRT4‐H161Y (0.5 mg mL^−1^) in HEPES buffer (40 mm HEPES, 1 mm MgCl₂, 1 mm dithiothreitol) in the presence or absence of 5 mm NAD (Sigma–Aldrich, St. Louis, MO, USA) at 37 °C for 1 h. Reaction products were analyzed by Western blot using antibodies listed in Table S5 (Supporting Information).

### Immunofluorescent Cytochemistry

Cells grown on glass coverslips were stained with MitoTracker Red CMXRos (Thermo Fisher Scientific, Waltham, MA, USA) for 30 min at 37 °C to label mitochondria, followed by fixation with ice‐cold acetone for 5 min at room temperature. After washing with 1×PBS three times, cells were permeabilized with 0.5 % Triton‐X‐100/PBS，and incubated with Rabbit anti‐SIRT4 (1:200) antibody (Sigma‐Aldrich, St. Louis, MO, USA) for 1 hour at 37 °C, followed by incubation with FITC‐conjugated goat anti‐rabbit antibody (Thermo Fisher Scientific) at 1:200 for 1 hour at 37 °C.

For SIRT4 and ENO1 co‐localization study, fixed cells were incubated with mouse anti‐ENO1 (1:200) and rabbit anti‐SIRT4 (1:200) (Sigma–Aldrich, St. Louis, MO, USA) antibodies in PBS for 1 h at 37 °C, followed by incubation with Alexa Fluor 594‐conjugated goat anti‐rabbit, and FITC‐conjugated goat anti‐mouse antibodies. Nuclei were counterstained with 4′,6‐diamidino‐2‐phenylindole (DAPI, 0.5 mg mL^−1^; Polysciences, Warrington, PA, USA) for 3 min. Samples were mounted in 90% glycerol/PBS containing 2.5% 1,4‐diazabicyclo(2,2,2)octane (DABCO) and visualized using a Leica SP5 confocal microscope (Leica, Wetzlar, Germany) equipped with a 63× oil immersion objective.

### RNA Extraction and Quantitative Real‐Time PCR

Total RNA was extracted from cells using TRIzol reagent (Invitrogen, Carlsbad, CA, USA), and was reverse transcribed into complementary DNA (cDNA) using the RevertAid First Strand cDNA Synthesis Kit (Thermo Fisher Scientific) according to the manufacturer's instructions.

Quantitative real‐time PCR (qRT‐PCR) was conducted using the SYBR Premix Ex Taq (Tli RNaseH Plus) Kit (Takara, Shiga, Japan) on an ABI PRISM 7500 Sequence Detector (Thermo Fisher Scientific, Waltham, MA, USA). Relative mRNA expression levels were calculated using the 2^‐ΔΔCT^ method, where ΔCT = CT (target gene) – CT (reference gene). *β‐actin* was used as the internal reference gene. The primer sequences used in this study are listed in Table S6 (Supporting Information).

### Surface Plasmon Resonance (SPR)

SPR experiments were performed using a Biacore T200 instrument (GE Healthcare, Chicago, IL, USA). Wild‐type ENO1, ENO1‐K358R, and ENO1‐K358Q proteins were diluted to 50 µg mL^−1^ in sodium acetate buffer (pH 5.0) and immobilized on a CM5 sensor chip (GE Healthcare, Chicago, IL, USA) via amine coupling to achieve a target density of 10 000 resonance units (RU). Binding interactions were measured and analyzed using the manufacturer's software.

### RNA Immunoprecipitation Sequencing

RNA immunoprecipitation assays were conducted using the Magna RIP^TM^ RNA‐Binding Protein Immunoprecipitation Kit (Millipore, Billerica, MA, USA) following the manufacturer's instructions. Protein A Magnetic beads (Sigma–Aldrich) were coated with 5 µg of anti‐ENO1 antibodies, and were incubated with cell lysates overnight at 4 °C. The precipitated RNA‐protein complexes were washed and treated with proteinase K to digest proteins. Co‐immunoprecipitated RNAs were extracted using phenol–chloroform methods, and were reverse transcribed into cDNA using the RevertAid First Strand cDNA Synthesis Kit (Thermo Fisher Scientific), followed by second‐strand cDNA synthesis with DNA polymerase I. Adaptor ligation and PCR amplification were performed to prepare the libraries. High‐throughput sequencing was carried out using the Illumina HiSeq 2500 platform by Novogene (Tianjin, China). Enrichment of specific RNA fragments was confirmed by real‐time PCR and normalized to input RNA. Primer sequences for small nucleolar RNA (snoRNA) quantification are listed in Table S6 (Supporting Information).

### Transcription and Biotinylation of SNORA25 In Vitro

RNA obtained from RIP assay was used as templates for in vitro transcription. A T7 promoter sequence was added to the 5′ end of the SNORA25 primer (Table S6, Supporting Information). Transcription was performed using the MAXIscript SP6/T7 Transcription Kit (Thermo Fisher Scientific, Waltham, MA, USA). The transcribed SNORA25 RNA was biotinylated using the Biotin RNA Labeling Mix (Roche, Mannheim, Germany) according to the manufacturer's instructions.

### RNA‐Sequencing and Data Analysis

Cultured cells were collected in TRIzol Reagent (Invitrogen, Carlsbad, CA, USA), and sent to Mingma Technologies (Shanghai, China) for mRNA purification, library preparation and sequencing. High‐throughput sequencing was performed on the Illumina NovaSeq 6000 platform to produce 150 bp paired‐end reads. Raw sequences were converted into fastq format files and removed the adapters with Trim galore software (v.0.6.7), and the clean reads were first aligned to the human 45S pre‐rRNA and unmapped reads were collected to align to the human genome assembly version (hg19) using the HISAT2 software (v.2.1.0). Mapped reads were used to quantify the number of genes by featureCounts program (v.2.0.1). The differentially expressed genes (DEGs) were defined using the DESeq2 package (v.1.24.0) with thresholds of FDR < 0.05 and |log_2_ (FoldChange)| > 1.5.

### CUT&Tag

The CUT&Tag assay was performed using the Hyperactive Universal CUT&Tag Assay Kit for Illumina (Vazyme, Nanjing, China) according to the manufacturer's protocol. Briefly, cells were harvested, counted, and centrifuged at 600 × *g* for 3 min at room temperature. Approximately 5 × 10^5^ cells were washed twice with Wash Buffer by gentle pipetting. Pre‐activated Concanavalin A‐coated magnetic beads were added to the cell suspension and incubated at room temperature for 15 min. The bead‐bound cells were then incubated with a primary antibody diluted in 50 µL Dig‐Wash buffer on a rotating platform overnight at 4 °C. Subsequently, a secondary antibody was added and incubated at room temperature for 1 h. The pA‐Tn5 adapter complex was added to the cells and incubated at room temperature for 1 h. Cells were resuspended in Tagmentation Buffer and incubated at 37 °C for 1 h. DNA was extracted using AMPure XP beads, and libraries were amplified. Finally, libraries were purified using the MiniElute Gel Extraction Kit (Qiagen, Hilden, NRW, Germany). Library concentration was quantified using the Qubit dsDNA HS Assay Kit (Thermo Fisher Scientific, Waltham, MA, USA), and sequencing was performed on an Illumina NovaSeq 6000 platform.

### Statistical Analysis

All data were analyzed using GraphPad Prism 9.5 software (San Diego, CA, USA). Tumorigenic frequency was calculated using extreme limiting dilution analysis at http://bioinf.wehi.edu.au/software/elda/. Differences were analyzed using unpaired two‐tailed *t*‐tests. A *p*‐value ≤ 0.05 was considered statistically significant.

### Ethical Statement

All animal experiments were conducted in accordance with the National Institutes of Health Guide for the Care and Use of Laboratory Animals and approved by the Peking University Cancer Hospital Animal Care and Use Committee.

## Conflict of Interest

The authors declare no conflict of interest.

## Author Contributions

M.L., X.Y., C.X., Q.S., and H.Z. contributed equally to this work. Z.Z., Y.X., and G.S. designed the study. M.L., Q.S., X.Y., J.L., and C.X. performed experiments. H.Z. and T.S. conducted bioinformatics analysis. Y.Z. provided technical consultation. M.L., Q.S., and Z.Z. wrote the manuscript. Z.Z. supervised the study. All authors have read and approved the article.

## Supporting information



Supporting Information

Supporting Table

Supporting Table

Supporting Table

Supporting Table

Supporting Table

Supporting Table

## Data Availability

The data that support the findings of this study are available in the supplementary material of this article.
