# Supplementary material for: SIRT4 Promotes Pancreatic Cancer Stemness by Enhancing Histone Lactylation and Epigenetic Reprogramming Stimulated by Calcium Signaling
Source: Adv Sci (Weinh). 2025 Apr 29;12(20):2412553. doi: 10.1002/advs.202412553 (PMC12120773; doi:10.1002/advs.202412553)
Supplement: Supplementary file 1 — Supporting Information [file ADVS-12-2412553-s006.docx]

**Supplementary information**

**Supplementary figures**

**
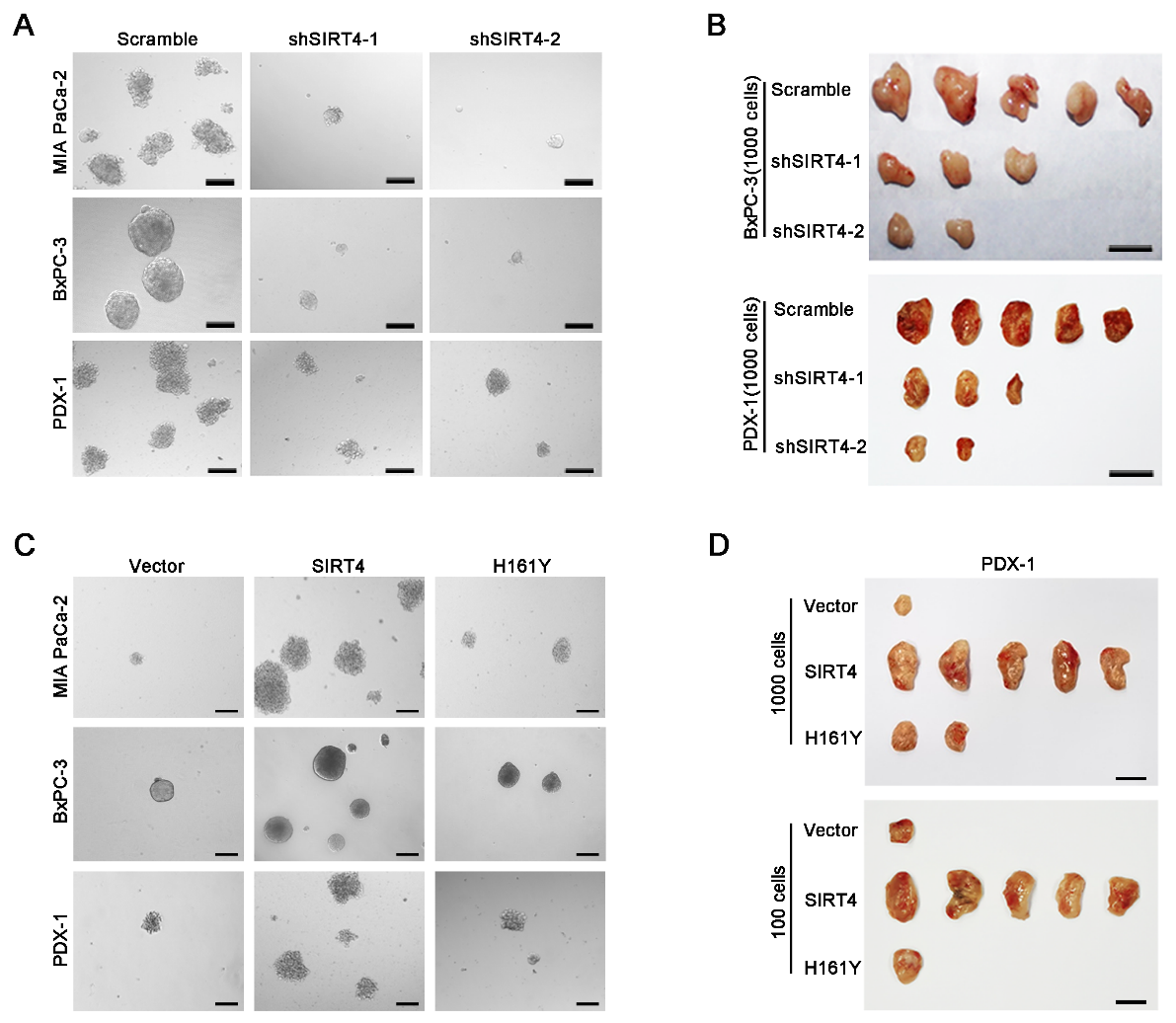
**

**Figure S1. SIRT4 plays a crucial role in the acquisition and maintenance of stem cell-like properties of PDAC cells.**

**A**. Representative phase contrast micrographs showing the spheroids formed by α2δ1^+^ cells sorted from the indicated sources following knockdown of SIRT4 by specific shRNAs. Scale bars, 100 μm. **B.** Photographs showing the dissected tumors formed by α2δ1^+^ cells sorted from the indicated sources following knockdown of SIRT4 by specific shRNAs. Scale bars, 1 cm. **C.** Representative phase contrast micrographs showing the spheroids formed by α2δ1^¯^ cells sorted from the indicated sources following overexpression of the indicated constructs. Scale bars, 100 μm. **D.** Photographs showing the dissected tumors formed by α2δ1^¯^ cells sorted from the indicated sources following overexpression of the indicated constructs. Scale bars, 1 cm.

**
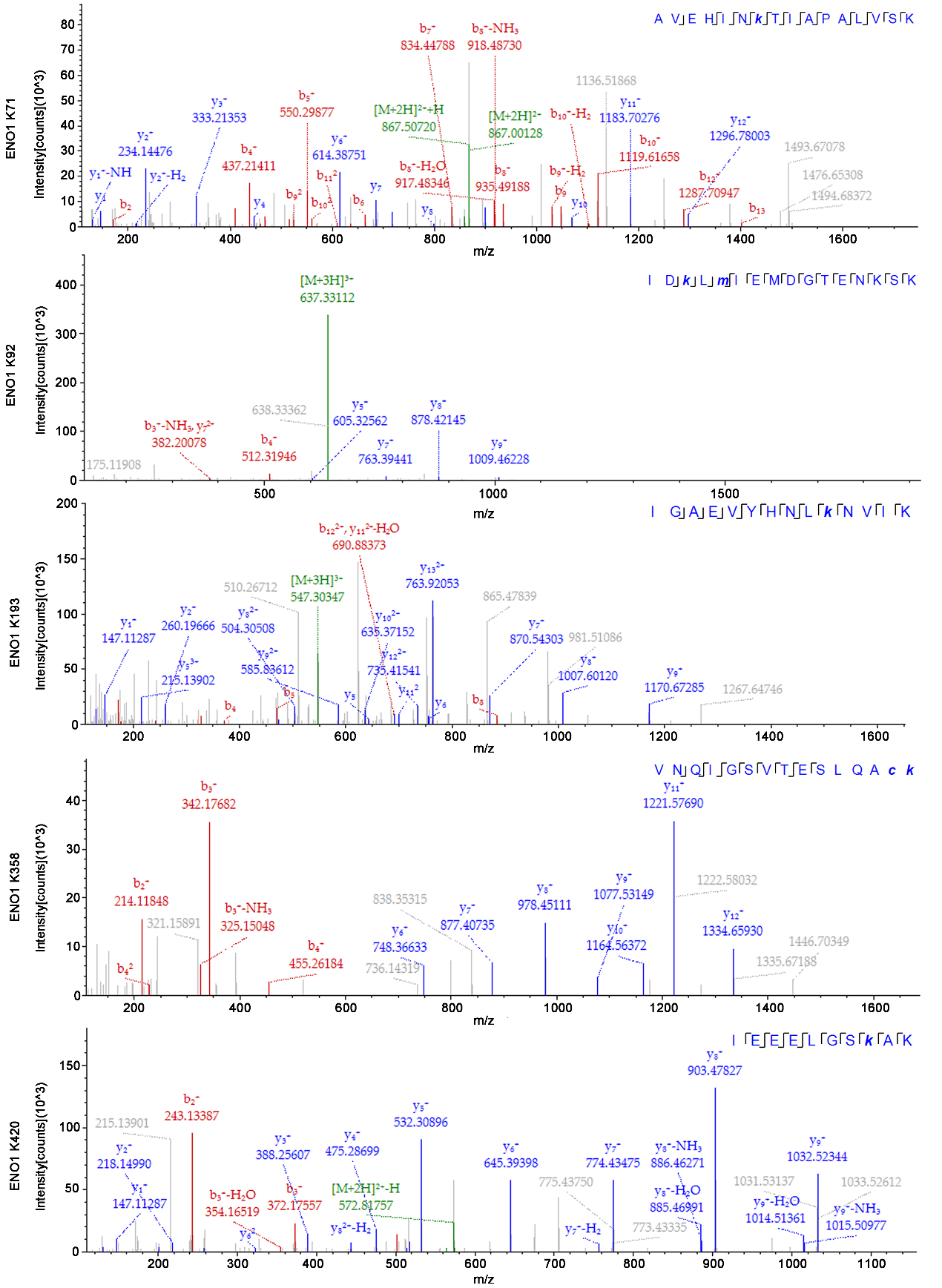
**

**Figure S2. The images of mass spectrometry peak of ENO1 acetylation.**

Mass spectrometry peak showing the acetylation of ENO1 K71, K92, K193, K358 and K420 sites in PANC-1 cells expressing the empty vector relative to SIRT4-overexpressed group.

**
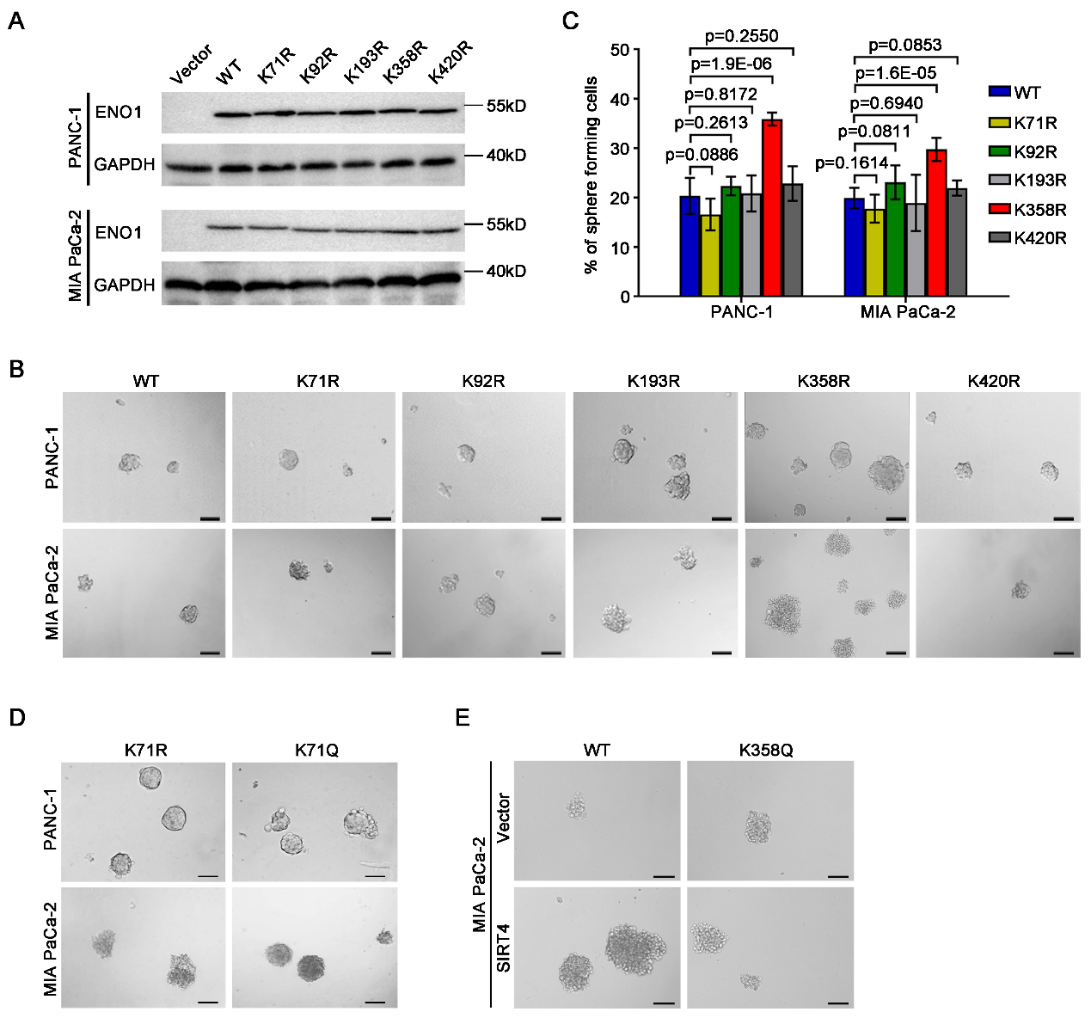
**

**Figure S3. The deacetylation of ENO1 at K358 promotes stem cell-like properties of PDAC cells.**

**A.** Western blot analysis of the expression of ENO1 in the indicated ENO1-knockout (ENO1-KO) cells overexpressing the indicated sgRNA-resistant constructs. **B.** Representative phase contrast micrographs showing the spheroids formed by the indicated ENO1-KO cells overexpressing the indicated sgRNA-resistant constructs. Scale bars, 100 μm. **C.** Histograms showing the spheroid formation efficiencies in the indicated ENO1-KO cells overexpressing the indicated sgRNA-resistant constructs. Cells were plated at 100 cells/well in 96-well plates (n=6). Data were the mean ± SD of three independent experiments and were analyzed by two-tailed Student’s *t*-test. **D.** Representative phase contrast micrographs showing the spheroids formed by the indicated ENO1-KO cells expressing the indicated sgRNA-resistant constructs. Scale bars, 100 μm. **E.** Representative phase contrast micrographs showing the spheroids formed by ENO1-KO MIA PaCa-2 cells expressing the indicated sgRNA-resistant constructs after SIRT4 overexpression. Scale bars, 50 μm.

**
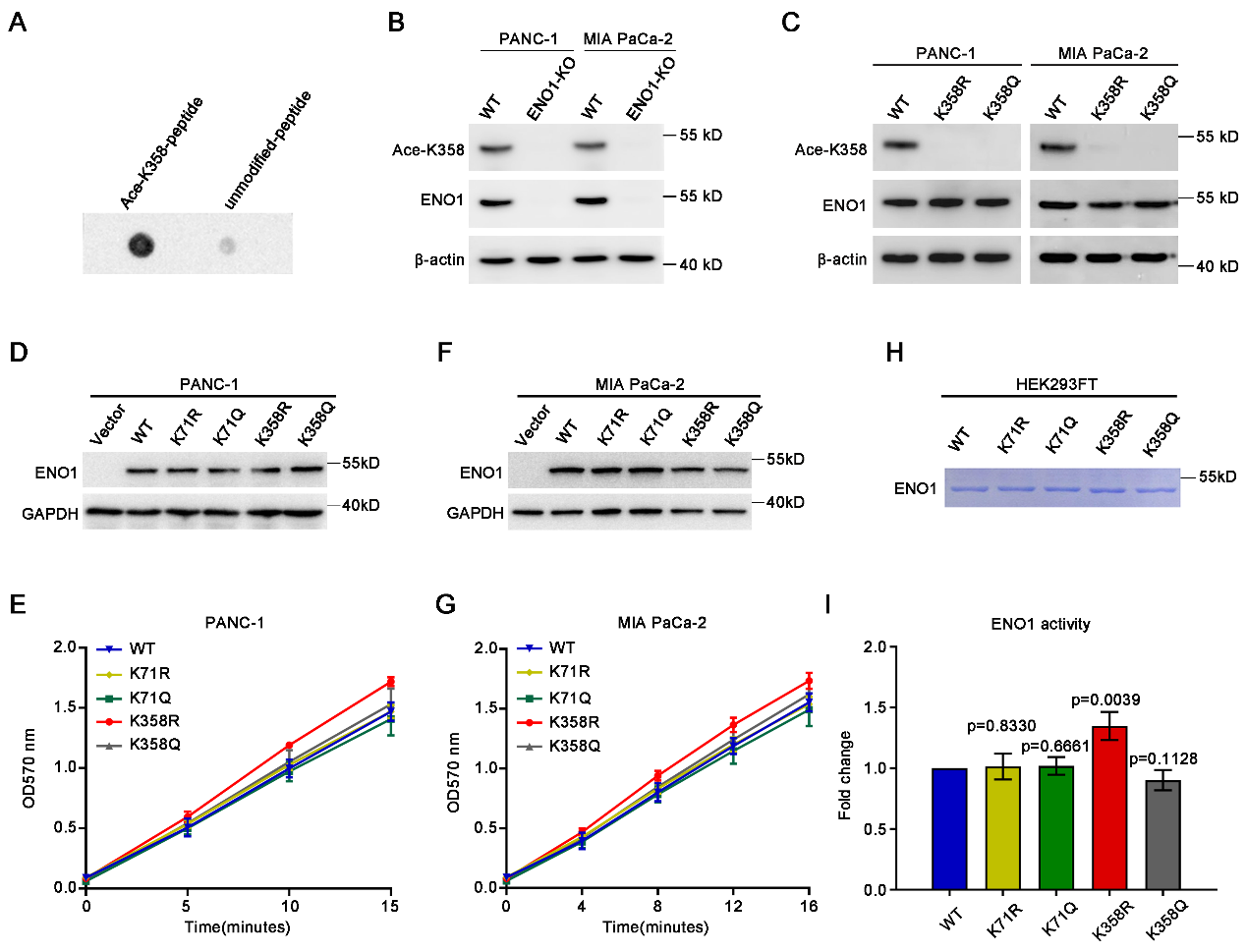
**

**Figure S4. Deacetylation of ENO1 at K358 enhances the enzymatic activity of purified ENO1.**

**A.** Characterization of the site-specific acetylation antibody against ENO1-K358. The acetylated ENO1 at K358 peptide or unmodified peptide were spotted onto a nitrocellulose membrane, and probed with the Ace-K358 antibody. **B.** Western blot indicating the specificity of acetylated K358 antibody in the indicated ENO1-knockout cells. **C.** Western blot analysis with the acetylated K358 antibody in the cell lysates from the indicated ENO1-knockout cells expressing the indicated ENO1 mutants. **D, F.** Western blot analysis showing the expression of ENO1 mutants in the ENO1-KO cells overexpressing the indicated constructs. **E, G.** Line graph showing the dynamic changes of the enzymatic activity of ENO1 in the indicated cells (n=3). **H.** SDS-PAGE analysis of purified ENO1 mutants expressed in HEK293FT cells. **I.** Histograms showing the enzymic activity of ENO1 protein purified with anti-FLAG resin from HEK293FT cells expressing the indicated ENO1 mutants (n=3). Data were presented as mean ± SD. Unpaired two-tailed Student’s *t*-test was used for statistical analysis.

**
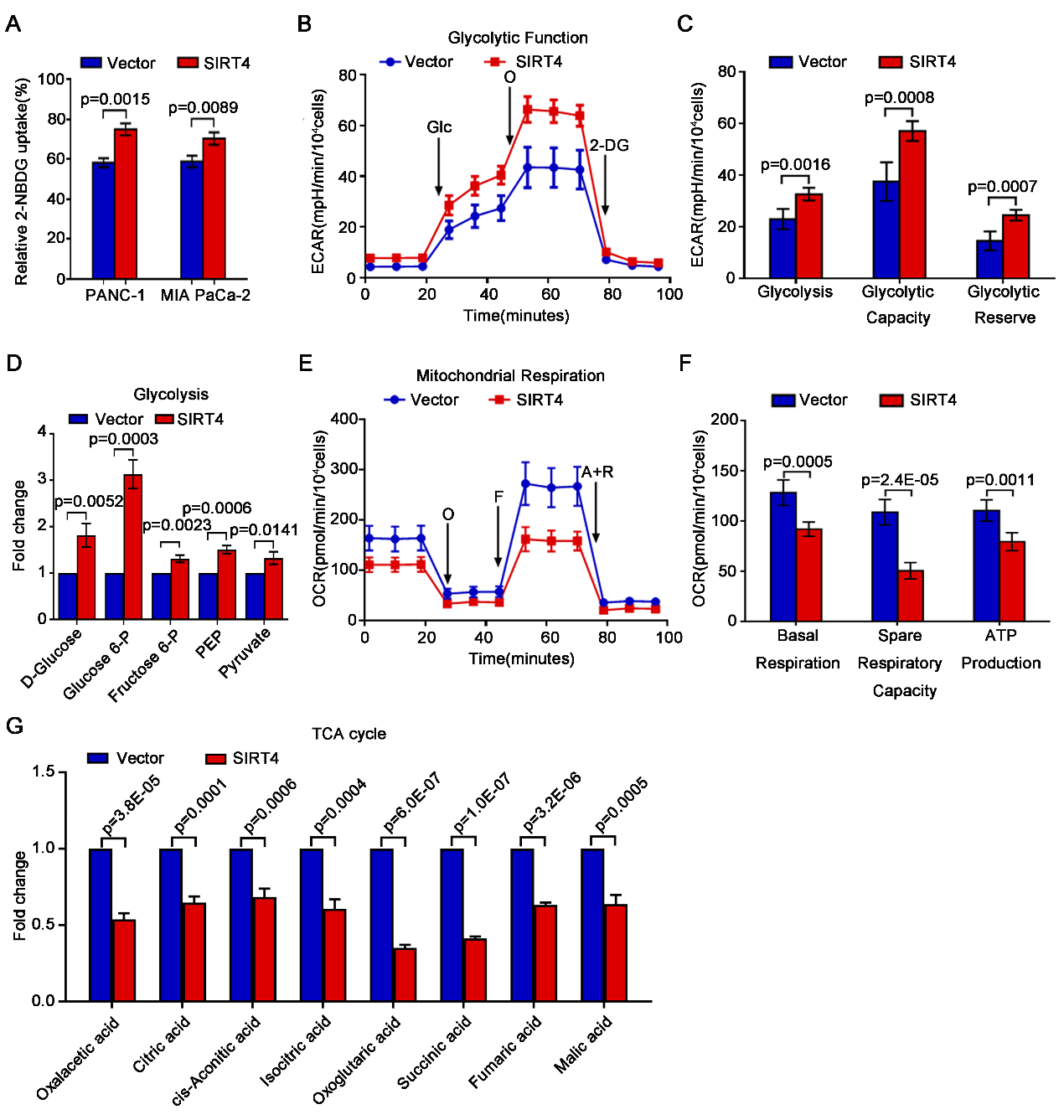
**

**Figure S5. SIRT4 promotes the glycolysis and inhibits mitochondrial respiration in pancreatic cancer cells.**

**A.** The glucose up-take capacity was determined in the indicated α2δ1^¯^cells following forced expression of SIRT4 (n=3). **B.** ECAR curve showing the changes of glycolysis of α2δ1^¯^ MIA PaCa-2 cells overexpressing empty vector alone and SIRT4 (n=5). Glucose (Glc), Oligomycin (O). **C.** Histogram showing the changes of glycolysis, glycolytic capacity, and glycolytic reserve in α2δ1^¯^ MIA PaCa-2 cells after SIRT4 overexpression (n=5). **D.** Non-targeted metabolomics mass spectrometry analysis indicating the changes of the indicated intermediate metabolites in glycolysis pathway after overexpressing SIRT4 in α2δ1^¯^ PANC-1 cells (n=3). **E.** OCR curve showing the changes of mitochondrial respiration of α2δ1^¯^ MIA PaCa-2 cells after forced expression of SIRT4 (n=5). Oligomycin (O), FCCP (F), Antimycin A (A), Rotenone (R). **F.** Histogram showing the changes of basal respiration, spare respiratory capacity, and ATP production following forced expression of SIRT4 in α2δ1^¯^ MIA PaCa-2 cells (n=5). **G.** Histogram showing the changes of the indicated intermediate metabolites of TCA cycle in α2δ1^¯^ PANC-1 cells infected with empty vector or SIRT4 lentiviruses (n=3). Data in **A**, **C**, **D**, **F** and **G** were presented as mean ± SD of three independent experiments. Unpaired two-tailed Student’s *t*-test was used for statistical analysis.

**
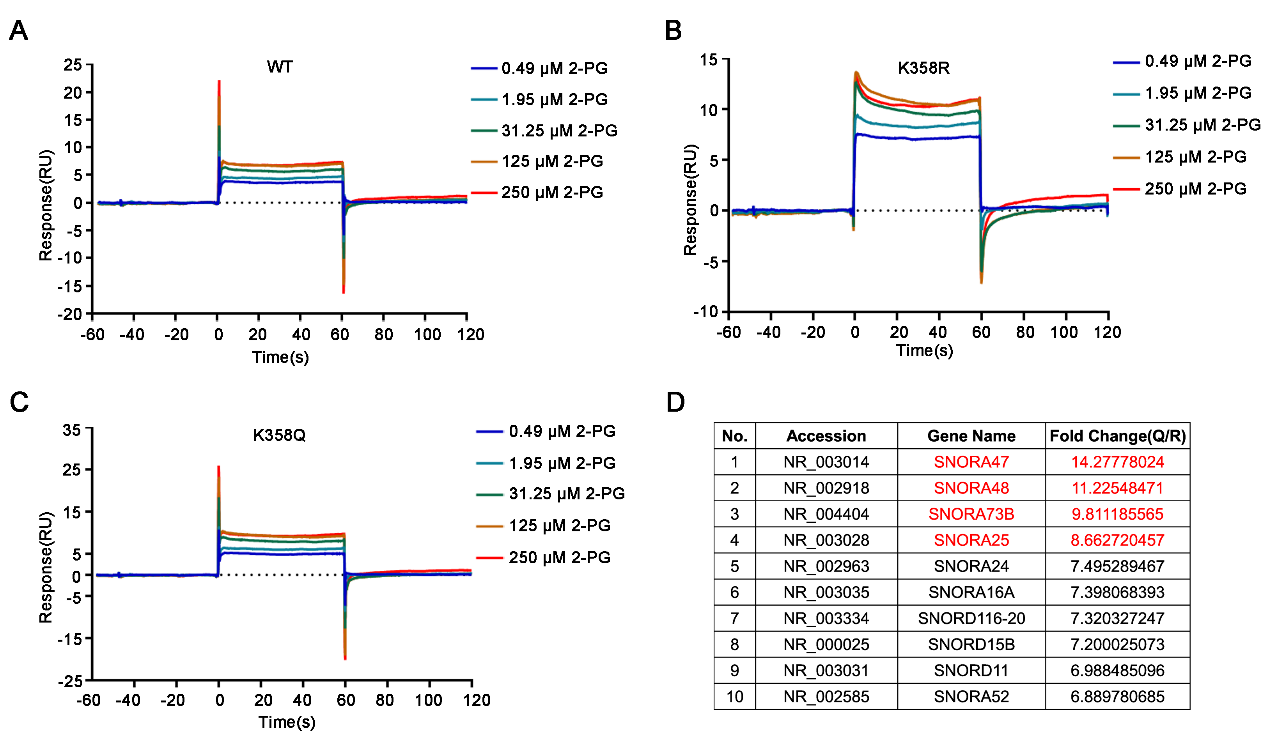
**

**Figure S6. The binding capacity of ENO1 mutants to 2-PG and SnoRNAs.**

**A - C.** SPR sensorgram profiles of ENO1 substrate 2-PG at gradient concentrations on a sensor chip immobilized with wild type ENO1, ENO1^K358R^, and ENO1^K358Q^ protein. SPR assays were performed at pH 4.5. **D.** Chart indicating the upregulated snoRNAs molecules (top 10) in ENO1^K358Q^ group compared with ENO1^K358R^ group in RIP-seq data.

**
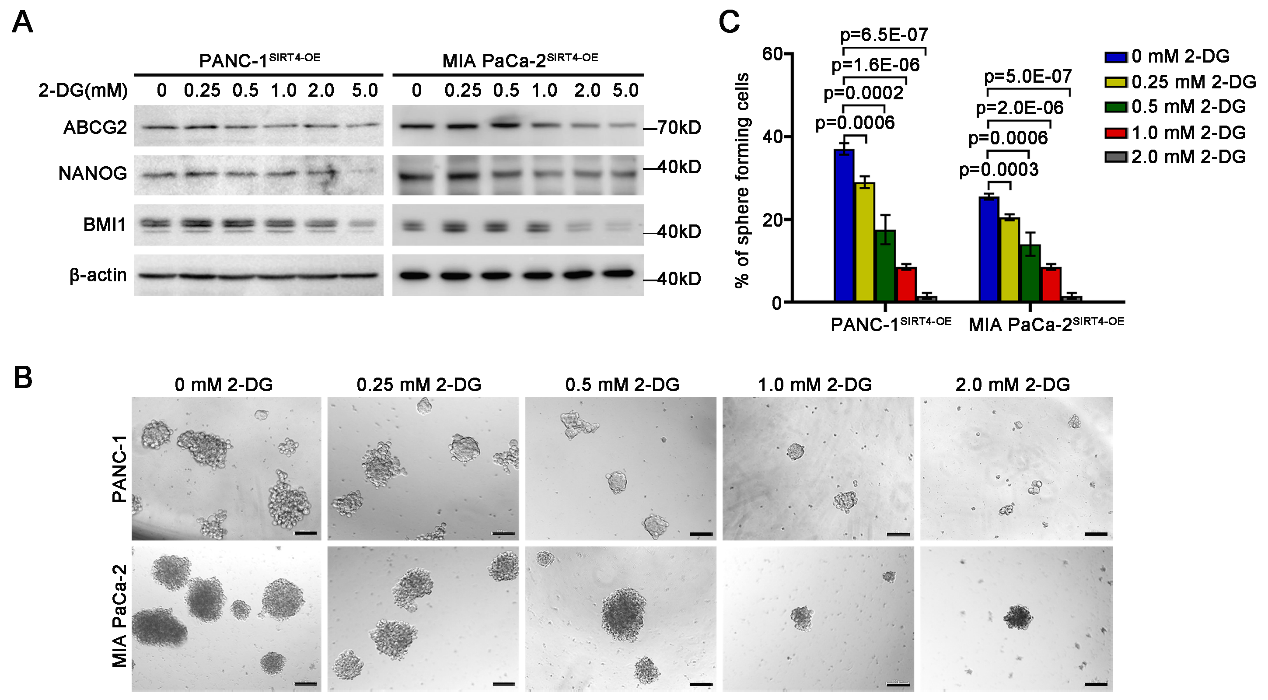
**

**Figure S7. Glycolysis inhibition attenuates the stem cell-like traits of PDAC cells expressing SIRT4.**

**A.** Western blot analysis showing the expression of the indicated molecules in the indicated SIRT4-overexpressing cells treated with different concentrations of 2-DG. β-actin was used as an internal reference. **B.** Representative micrographs of spheres formed by the indicated SIRT4-overexpressing cells after 2-DG treatment. Scale bars, 100 μm. **C.** Histogram showing the spheroid formation efficiencies of the indicated SIRT4-overexpressed cells treated with 2-DG (n=3). Data were the mean ± SD of three independent experiments. Unpaired two-tailed Student’s *t*-test was used for statistical analysis.

**Supplementary tables**

**Supplementary table 1. The tumorigenicity of SIRT4-depleted PDAC cells in NOD/SCID mice.**

| Groups | Tumor formation | | Frequency of tumorigenic | P value* |
| --- | --- | --- | --- | --- |
|  | 1000 | 100 | cells (95% CI) |  |
| PANC-1 Scramble | 5/5 | 4/5 | 1 / 62.1(1/ 185-1/20.9) |  |
| PANC-1 shSIRT4-1 | 2/5 | 2/5 | 1 / 1060.9 (1/ 3128 -1/359.8) | 2.75E-04 |
| PANC-1 shSIRT4-2 | 1/5 | 1/5 | 1/2457.9 (1/ 10394 -1/581.3) | 1.31E-05 |
| MIA PaCa-2 Scramble | 5/5 | 4/5 | 1 / 62.1(1/ 185-1/20.9) |  |
| MIA PaCa-2 shSIRT4-1 | 1/5 | 2/5 | 1 / 1615.9 (1/ 5534 -1/471.8) | 4.83E-05 |
| MIA PaCa-2 shSIRT4-2 | 1/5 | 0/5 | 1/9491.2 (1/ 67440 -1/1335.8) | 2.99E-08 |
| BxPC-3 Scramble | 5/5 | 4/5 | 1 /62.6 (1/ 185-1/21.4) |  |
| BxPC-3 shSIRT4-1 | 3/5 | 2/5 | 1 / 712 (1/ 1929 -1/263) | 1.50E-03 |
| BxPC-3 shSIRT4-2 | 2/5 | 1/5 | 1 /1445.5 (1/ 4728-1/442.2) | 9.63E-05 |
| PDX-1 Scramble | 5/5 | 5/5 | 1 /1 (1/ 125 -1/1) |  |
| PDX-1 shSIRT4-1 | 3/5 | 2/5 | 1 / 711 (1/ 1928 -1/262) | 3.32E-05 |
| PDX-1 shSIRT4-2 | 2/5 | 1/5 | 1 / 1445 (1/ 4728 -1/442) | 1.48E-06 |
| PDX-2 Scramble | 5/5 | 4/5 | 1 /62.6 (1/ 185 -1/21.4) |  |
| PDX-2 shSIRT4-1 | 3/5 | 1/5 | 1 / 921.2 (1/ 2618 -1/324.4) | 6.18E-04 |
| PDX-2 shSIRT4-2 | 1/5 | 0/5 | 1 / 4983.8 (1/ 35192 -1/706.2) | 2.28E-06 |

*Compared with respective Scramble group. Data were analyzed using the extreme limiting dilution analysis tool at <http://bioinf.wehi.edu.au/software/elda/>.

**Supplementary table 2. The tumorigenicity of SIRT4 mutated PDAC cells in NOD/SCID mice.**

| Groups | Tumor formation | | Frequency of tumorigenic | P value |
| --- | --- | --- | --- | --- |
|  | 1000 | 100 | cells (95% CI) |  |
| PANC-1 Vector | 2/5 | 1/5 | 1 / 1445 (1/ 4728 -1/442) |  |
| PANC-1 SIRT4 | 5/5 | 5/5 | 1 /1(1/125-1/1) | 1.48E-06 |
| PANC-1 SIRT4^H161Y^ | 2/5 | 2/5 | 1 / 1061 (1/ 3128 -1/360) | 0.689 |
| MIA PaCa-2 Vector | 1/5 | 0/5 | 1 / 4983 (1/ 35191 -1/706) |  |
| MIA PaCa-2 SIRT4 | 5/5 | 5/5 | 1 /1(1/125-1/1) | 2.58E-08 |
| MIA PaCa-2 SIRT4^H161Y^ | 2/5 | 1/5 | 1 / 1445 (1/ 4728 -1/442) | 0.249 |
| PDX-1 Vector | 1/5 | 1/5 | 1 / 2458 (1/ 10394 -1/581) |  |
| PDX-1 SIRT4 | 5/5 | 5/5 | 1 /1(1/125-1/1) | 1.63E-07 |
| PDX-1 SIRT4^H161Y^ | 2/5 | 1/5 | 1 / 1445 (1/ 4728 -1/442) | 0.559 |
| PDX-2 Vector | 1/5 | 1/5 | 1 / 2458 (1/ 10394 -1/581) |  |
| PDX-2 SIRT4 | 5/5 | 5/5 | 1 /1(1/125-1/1) | 1.63E-07 |
| PDX-2 SIRT4^H161Y^ | 2/5 | 1/5 | 1 / 1445 (1/ 4728 -1/442) | 0.559 |

*Compared with respective control cells expressing vector alone. Data were processed using the extreme limiting dilution analysis tool at <http://bioinf.wehi.edu.au/software/elda/>.

**Supplementary table 3. The tumorigenicity of ENO1 mutated PDAC cells in NOD/SCID mice.**

| Groups | Tumor formation | | Frequency of tumorigenic | P value* |
| --- | --- | --- | --- | --- |
|  | 1000 | 100 | cells (95% CI) |  |
| PANC-1 ENO1^WT^ | 5/5 | 1/5 | 1 / 288 (1/ 835 -1/99.1) | - |
| PANC-1 ENO1^K358R^ | 5/5 | 5/5 | 1 /1(1/125-1/1) | 0.0049 |
| PANC-1 ENO1^K358Q^ | 5/5 | 2/5 | 1 / 184 (1/ 609 -1/55.9) | 0.601 |
| PANC-1 ENO1^K71R^ | 3/5 | 3/5 | 1 / 571 (1/ 1519 -1/215) | 0.335 |
| PANC-1 ENO1^K71Q^ | 4/5 | 1/5 | 1 / 580 (1/ 1543 -1/218.1) | 0.348 |
| MIA PaCa-2 ENO1^WT^ | 5/5 | 0/5 | 1 / 417 (1/ 1120 -1/155.3) | - |
| MIA PaCa-2 ENO1^K358R^ | 5/5 | 4/5 | 1 /62.1(1/185-1/20.9) | 0.0225 |
| MIA PaCa-2 ENO1^K358Q^ | 4/5 | 4/5 | 1 / 580.1 (1/ 1543 -1/218.1) | 0.657 |
| MIA PaCa-2 ENO1^K71R^ | 4/5 | 1/5 | 1 / 580.1 (1/ 1543 -1/218.1) | 0.657 |
| MIA PaCa-2 ENO1^K71Q^ | 3/5 | 0/5 | 1 / 1268.3 (1/ 3956 -1/406.7) | 0.161 |

*Compared with the respective control cells expressing wild type ENO1 (WT). Data were processed using the extreme limiting dilution analysis tool at <http://bioinf.wehi.edu.au/software/elda/>

**Supplementary table 4. shRNA sequences used in this study.**

| shRNA | shRNA sequence (5’ to 3’) |
| --- | --- |
| Scramble shRNA | GATCCGCGAGAAGCGCGATCACATGTTCAAGAGACATGTGATCGCGCTTCTCGTTTTTG |
| shSIRT4-1 | GATCCCCCGATTGCAATACTGAACATCTTCCTGTCAGAATGTTCAGTATTGCAATCGGGTTTTTG |
| shSIRT4-2 | GATCCCCGTGCTCGAAAGCCTCCATTCTTCCTGTCAGAAATGGAGGCTTTCGAGCACGGTTTTTG |

**Supplementary table 5. Information for primary antibodies used in Western Blot.**

| Name | Vendor | Catalog No. | Dilution |
| --- | --- | --- | --- |
| α2δ1 | Abcam | ab2864 | 1:2000 |
| CaMK2D | Abcam | ab181052 | 1:2000 |
| SIRT4 | Sigma | HPA029691 | 1:2000 |
| ENO1 | Sigma | WH0002023M1 | 1:4000 |
| ABCG2 | Abcam | ab207732 | 1:2000 |
| BMI1 | Abcam | ab126783 | 1:4000 |
| SOX2 | Cell Signaling Technology | 3579S | 1:2000 |
| NANOG | Cell Signaling Technology | 3580S | 1:2000 |
| Acetylated-Lysine | Cell Signaling Technology | 9441S | 1:2000 |
| Acetylated-ENO1(Lys358) | Willget biotech | - | 1:1000 |
| HA | Cell Signaling Technology | 3724s | 1:4000 |
| FLAG | OriGene | TA50011 | 1:4000 |
| Lactylated Histone3(Lys9) | PTM BIO | PTM-1419RM | 1:3000 |
| Lactylated Histone3(Lys14) | PTM BIO | PTM-1414RM | 1:3000 |
| Lactylated Histone3(Lys18) | PTM BIO | PTM-1406RM | 1:3000 |
| Lactylated Histone3(Lys23) | PTM BIO | PTM-1413RM | 1:3000 |
| Histone3 | Beyotime | AH433-1 | 1:3000 |
| Lactylated Histone4(Lys8) | PTM BIO | PTM-1415RM | 1:3000 |
| Lactylated Histone4(Lys16) | PTM BIO | PTM-1417RM | 1:3000 |
| Histone4 | Cell Signaling Technology | 13919S | 1:5000 |
| GAPDH | Bioworld | AP0063 | 1:10000 |
| β-actin | Cell Signaling Technology | 3700S | 1:5000 |

**Supplementary table 6. Primer sequences used in this study.**

| Primer | Sense (5’ to 3’) | Anti-sense (5’ to 3’) |
| --- | --- | --- |
| CACNA2D1 | GCTATTCACGGATGGAGGAGAAG | CCATCCACTGAATAGGTCCTCTG |
| CAMK2D | ACACGGTGACTCCTGAAGCCAA | GTCTCCTGTCTGTGCATCATGG |
| SIRT4 | GTGGATGCTTTGCACACCAAGG | GGTTCAGGACTTGGAAACGCTC |
| ABCG2 | GTTCTCAGCAGCTCTTCGGCTT | TCCTCCAGACACACCACGGATA |
| BMI1 | GGTACTTCATTGATGCCACAACC | CTGGTCTTGTGAACTTGGACATC |
| SOX2 | GCTACAGCATGATGCAGGACCA | TCTGCGAGCTGGTCATGGAGTT |
| β-actin | CACTCTTCCAGCCTTCCTTCC | ACAGCACTGTGTTGGCGTAC |
| SNORA48 | TGTCCCTGACCTGGGTAGAGT | CTATGTGTCCAAGGCATGAAC |
| SNORA73B | TCCAACGTGGATACCCTGGGAGGTCACTC | ATATGTTTCCTGCATGGTTTGTCTC |
| SNORA25 | CATTTCAAAGAGGGCTTATGAGGCTGTGAAAC | ATATCTCCTCAGGAAAACATAGCACC |
| SNORA25-T7 | TAATACGACTCACTATAGGGAGAgggtcatttcaaagagggcttatgaggctgtg | ttttatatctcctcaggaaaacatagcacc |
